# Tocilizumab degradation via photo-catalytic ozonation process from aqueous

**DOI:** 10.1038/s41598-023-49290-z

**Published:** 2023-12-16

**Authors:** Jamal Mehralipour, Hesam Akbari, Amir Adibzadeh, Hamed Akbari

**Affiliations:** 1 Health Research Center, Lifestyle Institute, Baqiyatallah University of Medical Sciences, Tehran, Iran; 2 Department of Environmental Health Engineering, Faculty of Health, Baqiyatallah University of Medical Sciences, Tehran, Iran

**Keywords:** Environmental sciences, Chemistry

## Abstract

Following the advent of the coronavirus pandemic, tocilizumab has emerged as a potentially efficacious therapeutic intervention. The utilization of O_3_-Heterogeneous photocatalytic process (O_3_-HPCP) as a hybrid advanced oxidation technique has been employed for the degradation of pollutants. The present study employed a solvothermal technique for the synthesis of the BiOI-MOF composite. The utilization of FTIR, FESEM, EDAX, XRD, UV–vis, BET, TEM, and XPS analysis was employed to confirm the exceptional quality of the catalyst. the study employed an experimental design, subsequently followed by the analysis of collected data in order to forecast the most favorable conditions. The purpose of this study was to investigate the impact of several factors, including reaction time (30–60 min), catalyst dose (0.25–0.5 mg/L), pH levels (4–8), ozone concentration (20–40 mMol/L), and tocilizumab concentration (10–20 mg/L), on the performance of O_3_-HPCP. The best model was discovered by evaluating the F-value and P-value coefficients, which were found to be 0.0001 and 347.93, respectively. In the given experimental conditions, which include a catalyst dose of 0.46 mg/L, a reaction time of 59 min, a pH of 7.0, and an ozone concentration of 32 mMol/L, the removal efficiencies were found to be 92% for tocilizumab, 79.8% for COD, and 59% for TOC. The obtained R^2^ value of 0.98 suggests a strong correlation between the observed data and the predicted values, indicating that the reaction rate followed first-order kinetics. The coefficient of synergy for the degradation of tocilizumab was shown to be 1.22. The catalyst exhibited satisfactory outcomes, but with a marginal reduction in efficacy of approximately 3%. The sulfate ion (SO_4_^2−^) exhibited no influence on process efficiency, whereas the nitrate ion (NO_3_^−^) exerted the most significant impact among the anions. The progress of the process was impeded by organic scavengers, with methanol exhibiting the most pronounced influence and sodium azide exerting the least significant impact. The efficacy of pure BiOI and NH_2_-MIL125 (Ti) was diminished when employed in their pure form state. The energy consumption per unit of degradation, denoted as EEO, was determined to be 161.8 KWh/m^3^-order.

## Introduction

China's Wuhan region was affected by acute atypical respiratory disease in December 2019. The disease spread rapidly to other areas from world. The cause of the epidemic was soon discovered to be a novel coronavirus. Because of its high homology with SARS-CoV, this novel virus has been named SARS-CoV2^1^. The World Health Organization (WHO) proclaimed the coronavirus disease 19 (COVID-19) pandemic. Approximately 200 countries and territories have reported COVID-19, affecting numerous people worldwide. According to reports, a wide range of agents and drugs were used to treat this disease worldwide, including antiviral, antibacterial, antimalarial, immunomodulatory, angiotensin II receptor blocker, Bradykinin B2 receptor antagonist, corticosteroids, anthelmintic, antiprotozoal, H2 blocker and anticoagulant^2^. The Food and Drug Administration (FDA) has granted approval to tocilizumab as a pharmaceutical intervention for the management of juvenile idiopathic arthritis, rheumatoid arthritisas well as chimeric antigen receptor (CAR)-cell-induced cytokine release syndrome (CRS). Here, it is an antibody that targets the Interleukin-6 receptor. The competitive inhibitor tocilizumab inhibits signaling mediated by IL-6. Overexpression of interleukin 6 is associated with chronic inflammation and autoimmunity due to its pathological effects on inflammation and immunity. Tocilizumab inhibits inflammatory responses by binding to interleukin-6 receptors, interrupting cellular signal transduction pathways^3^. As it has been proven in previous researches, the use of medicinal compounds leads to these compounds' presence in the environment, especially aquatic environments. Medicinal compounds are found in the environment in their original or modified form at different concentrations^4^. Therefore, medicinal compounds specific to the treatment of corona virus during the pandemic condition was predictable. The primary sources of pharmaceutical chemicals in the environment include home usage, pharmaceutical industry and hospital waste, and unintentional drug releases^5^. The presence of medicinal compounds in their parents and metabolized form causes potential risks that have been mentioned in previous studies. Medicinal compounds are in the group of compounds resistant to decomposition and, because of their complex structure, they face a challenge in common treatment processes^6^. The specific advantages and disadvantages can vary depending on the type of treatment method employed and the characteristics of the wastewater being treated. The selection of the most appropriate treatment method should consider factors such as the scale of operation, regulatory requirements, and the specific contaminants present in the wastewater^7^. Organic pollutants, can be rapidly oxidized and completely degraded using chemical methods^8^. The best way to treat organic wastewater is to use advanced oxidation processes (AOPs). AOPs are advantageous because of their high mineralization efficiency, simple operation, small footprint, high rate in oxidation, and lack of final hazardous pollution. Various AOPs can degrade organic pollutants by generating highly active radicals, including photocatalytic reactions, Fenton, sonochemical oxidation, electrochemical oxidation reactions, and catalytic ozonation^7^. Organic pollutants are oxidized and mineralized by AOPs, which produce reactive oxygen species (ROS_s_) such as hydroxyl radicals (OH^·^), superoxide radicals (O_2_^·−^), sulfates (SO_4_^·−^) and non-radicals like H_2_O_2_ and ^1^O_2_ that have oxidation and reduction potential (ORP) above 2 eV^9–11^. Due to its stability, low toxicity, affordability, and potent oxidative capabilities, heterogeneous photocatalysis of semiconductors have become one of the most important, environmentally sound, and widely applicable technologies^12^. This is because the photocatalyst completely mineralizes the pollutant without producing any hazardous byproducts^13^. In actuality, oxidation–reduction processes are used in heterogeneous photocatalysis to convert photons that are absorbed by semiconductors into chemical energy. Through the generation of highly oxidizing free radicals, excited catalysts are able to directly degrade contaminants that are adsorbed on their surfaces^14^. A study of the heterogeneous photocatalysis process (HPCP) focused on introducing new semiconductor materials as a photocatalyst. Several semiconductors, including MOFs, g-C_3_N_4_, and metal family oxides with slim band gaps, were used to extend the response spectrum to the visible spectrum^15^. UV-C activation is used to activate classic semiconductors like TiO_2_, CdO, CdSe, and ZnO. Solar radiation can't be used as a source of energy for these classic semiconductors due to their wide band gaps. It is activated by ultraviolet radiation, which represents less than 4% of solar radiation, and for these reasons, their use is limited^16^. Increasingly, metal–organic frameworks (MOFs) have been attracting attention for their ability to remove pollutants via photocatalysis and adsorption mechanism. Recent environmental studies have investigated various MOF-based photocatalysts. NH_2_-MIL125 (Ti) is a Ti-based amino-functioned MOF chosen for its non-toxicity, photo/water stability, and visible-light absorption^17^. As a result, MOFs can be restricted from being used as independent photocatalysts due to low migration efficiency, low charge separation, and poor conductivity. Several approaches have been suggested with the aim of enhancing the photocatalytic characteristics of MOFs. One of these methods is heterojunction structures^18^. Semiconductors based on bismuth are regarded as eco-friendly, stable, and nonpoisonous photocatalysts with high visible light absorbency. BiOI is a superior photocatalyst due to its layered structure, unique electrical characteristics, and suitable band gap (BG = 1.62–1.93 eV). In spite of this, BiOI has limited application due to much carrier recombination. In heterojunctions, charge separation and hole recombination can be reduced, thus overcoming this disadvantage^19^. NH_2_-MIL125 (Ti) serves as an excellent support material for BiOI due to its favorable properties, including high surface area and porosity. These characteristics facilitate the effective adsorption of BiOI nanoparticles, thereby promoting interfacial contact between BiOI and NH_2_-MIL125 (Ti) and enhancing charge transfer and separation efficiency. Notably, NH_2_-MIL125 (Ti) exhibits exceptional electron transfer properties attributed to its intrinsic electronic structure and the presence of amino functional groups. This enables efficient electron transfer from BiOI to NH_2_-MIL125 (Ti), effectively reducing charge recombination and further enhancing charge separation efficiency^20^. Moreover, the combination of BiOI and NH_2_-MIL125 (Ti) displays synergistic effects, resulting in improved photocatalytic performance and enhanced charge separation efficiency. The proper alignment of energy levels between BiOI and NH_2_-MIL125 (Ti) is crucial for facilitating efficient charge separation. Furthermore, NH_2_-MIL125 (Ti)'s stability and durability make it an ideal support material for BiOI, ensuring structural integrity and preventing the aggregation or degradation of BiOI nanoparticles. Consequently, this characteristic guarantees sustained photocatalytic performance and high charge separation efficiency over extended operational periods^21^. Numerous endeavors have been undertaken to hinder the deactivation of photocatalysis and enhance the performance of HPCP. The ozone enhanced photocatalytic oxidation process has garnered significant scrutiny due to its resilient capability in enhancing the efficacy of HPCP when eliminating organic compounds, commonly referred to as O_3_-HPCP^22^. Ozone is essential to the breakdown of organic matter because, in comparison to other processes, it is more affordable, ecologically benign, easily obtainable, and has a simple operating technique^23^. In this study, it was found that O_3_-HPCP increases reactive oxygen species, leading to faster mineralization rates of resistant organic compounds. O_3_-HPCP uses free radicals with higher ORP_s_ than ozone. Tocilizumab, the target pollutant, is broken down in O_3_-HPCP near the catalyst surface and reaction media^24^. Previous studies such as Liu and coworkers^25^ and Zhu and coworkers^26^ show the use of O_3_-HPCP has been used in the last few years to remove resistant organic compounds. Given the global prevalence of the coronavirus pandemic in recent years, and its continued presence in some countries necessitating the use of medications containing tocilizumab compounds, it is imperative to offer effective processes for treating wastewater containing these compounds. This study represents the inaugural investigation into the efficacy of O_3_-HPCP for the purpose of assessing its ability to removal the tocilizumab. Based on the explanations provided, the objectives of this research are divided into three parts. (1): Synthesis and characterization of BiOI-NH_2_-MIL125(Ti) (in abbravation BiOI-MOF) as a novel Co-MOF, (2): Optimization of parameters of O_3_-HPCP for the degradation of tocilizumab which was found in different concentrations during the corona outbreak in water environments such as hospital and pharmaceutical wastewater, and (3): Supplementary study on COD and TOC decreasing, reaction kinetics, synergist of mechanisms, catalyst's reusability, inorganic co-existing and organic radical scavenger’s effects, and energy consumption.

## Method and materials

### Materials and reagents

All chemical was purchased from trustworthy suppliers. The analytical grade samples did not require any further purification. Potassium iodide [KI], bismuth nitrate pentahydrate [Bi(NO_3_)_3_·5H_2_O], ethylene glycol [(CH_2_OH)_2_], N, N dimethylformamide [C_3_H_7_NO], tetrabutyl titanate [C_16_H_36_O_4_Ti], 2-aminoterephthalic acid [C_8_H_7_NO_4_], tocilizumab, sodium hydroxide [NaOH], benzoquinone (BQ), sodium bicarbonate [NaHCO_3_], oxalic acid [C_2_HO_4_], sodium nitrate [NaNO_3_], acetonitrile [C_2_H_2_N], trifluoroacetic acid (C_2_HF_3_O_2_), isopropanol (C_3_H_2__8_O), and methanol [CH_3_OH]. De-ionized water, with a resistivity of 16.25 MΩ/cm, was employed in the formulation of solutions.

### Development of a catalyst

#### Construction of NH_2_-MIL125(Ti)

As reported in previous literature, the solvothermal technique was used to create NH_2_-MIL-125 (Ti) with some adjustments to the amount of substances^27,28^. A solution consisting of 33 mL N, N-dimethylformamide (DMF), 4 mL of methanol, 1.55 g of 2-aminoterephthalic acid, and 2.2 mL of tetrabutyl titanate was mixed for 40 min at room temperature in an ultrasonic bathroom. Using a Teflon-lined autoclave allowed the mixture solution to be maintained at 180 °C for 60 h. The procedure was completed with the filtration of the suspension, followed by multiple rinsing with methanol and DMF, and finally drying in an electric furnace at 75 °C.

#### Construction of BiOI-NH_2_-MIL125(Ti)

The literature was used to create BiOI-MOF, with a few modifications in the quantity of materials^27^. Ultrasonic waves were used to dissolve 2 g of MOF in 40 cc deionized water with KI (1.2 mmol) for 30 min. The solution was combined with 13 mL of ethylene glycol and then delicately diluted with Bi(NO_3_)_3_.5H_2_O. The suspension was stirred intensively in a water bath of approximately 80°C for a duration of 30 min. The catalyst was sifted during refinement, washed using ethanol three times, and rinsed with distilled water, and then heated to 80°C.

#### Construction of BiOI

A comparative analysis was conducted to examine the physical, chemical, and other distinguishing features of BiOI, MOF and BiOI-MOF. Pure BiOI was synthesized without the presence of MOF in the solution.

#### Characterization

An FT-IR spectrophotometer (Sipotlight 220i FT-IR Microscopy Systems) was used to analyze the molecular composition of MOF, BiOI, and BiOI-MOF. The Rigaku/ZSX Primus 500 XRD diffractometer was used to conduct X-ray diffraction analysis. Average sample sizes were calculated using Scherrer's equation (Eq. 1)^29^.

1 $$D=\frac{(K.\lambda )}{(\beta .cos\theta )}$$

Here, the crystal sample size is represented by D, λ symbolizes the angle of diffraction, K represents a dimensionless constant, β represents the FWHM (full with half maximum) of the diffraction peak, and θ refers to the angle of diffraction.

The UV–visible spectrum (UV–Vis DRS) was analyzed to investigate the optical properties and structural characteristics using the Agilent Cary 60 spectrophotometer. Band gap energy was determined with the assistance of the Tauc equations (Eqs. 2,3)^30^.

2 $$\alpha hv={A\left(hv-Eg\right)}^{1/2}$$

3 $$\alpha hv={A\left(hv-Eg\right)}^{2}$$

where α is coefficient of absorption, v is light frequency, A is a constant parameter, hʋ is the photon energy of the incident photons, h is Planck's constant, and Eg is the band-gap of sample. To determine the band-gap energy of BiOI, a direct band-gap semiconductor, (Eq. 2) was applied. Similarly, for MOF, an indirect band-gap semiconductor, (Eq. 3) was utilized. The samples' morphology was surveyed using a FE-SEM (UN41219SEM) operating at a vacuum of ≥ 1.2 × 10–4 mbar. The utilization of energy dispersive spectroscopy (EDS) was employed in order to examine the elemental mapping as well as the integrity of the samples. The acquisition of a transmission electron microscopy image (TEM) was achieved by applying a stimulating voltage of 100 kV using the Shimadzu JEM-1200 EX. The nitrogen adsorption at 77 K allowed for the determination of the volume, pore size, and surface area distribution of BiOI-MOF. The samples underwent a process of degassing in their original position for a duration of 12 h at a temperature of 200°C, while a vacuum was present. The technique utilized for the determination of surface areas in the context of the linear correlation between p/p0 and surface areas was the Brunauer–Emmet–Teller (BET) technique. Analysis of the XPS characterizations was conducted with an ESCALAB250XI electron spectrometer.

### Installing O_3_-HPCP

**Figure 1:**
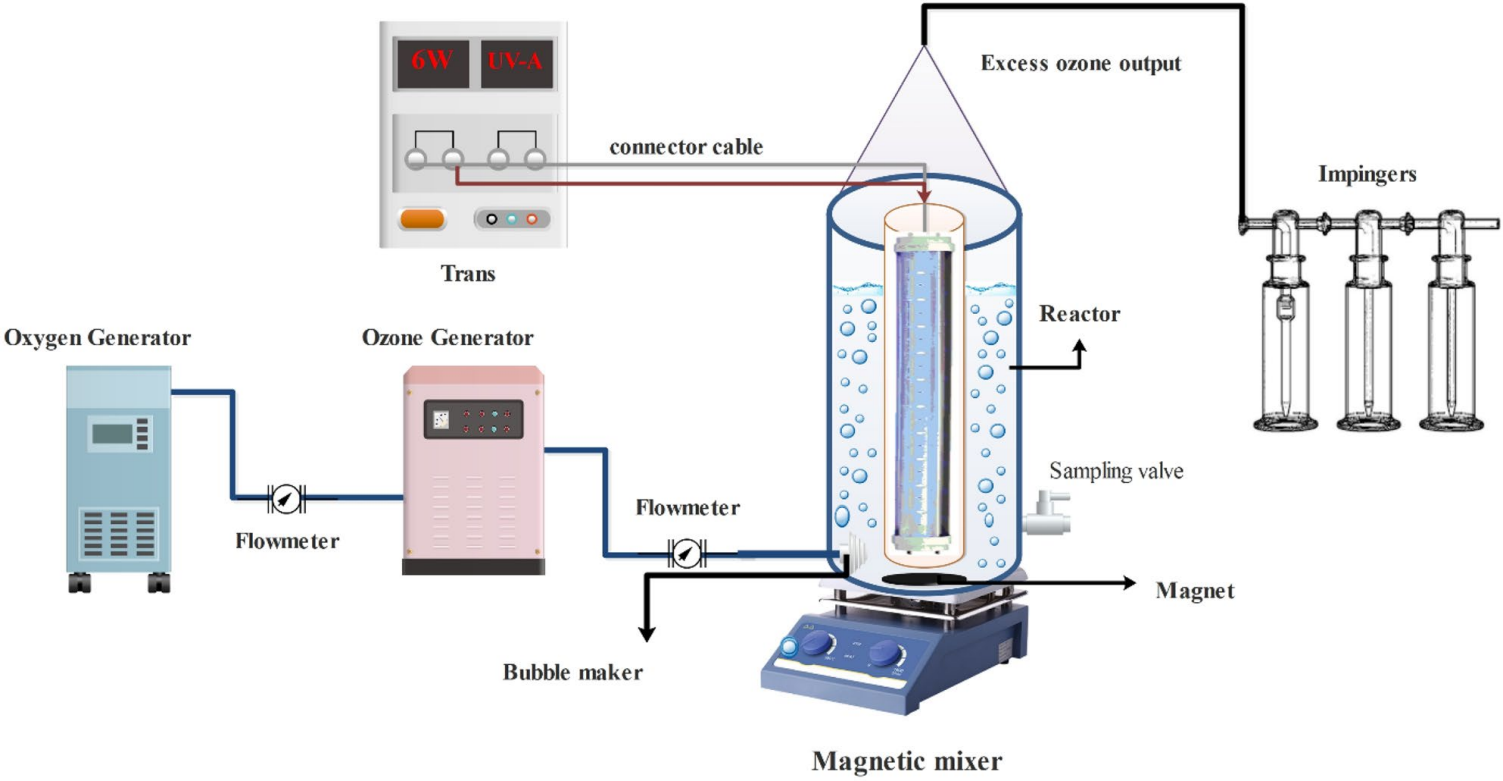
Diagram of the O_3_-HPCP.

A cylindrical unit measuring 600 mL, characterized by a diameter of 10 cm and a height of 20 cm, was employed for the O3-HPCP experiments. A quartz sheath (4.5 × 16 cm) was positioned centrally in a horizontal orientation (Fig. 1). The experiments were conducted utilizing a batch system under the conditions of an ambient temperature (25 ± 2 °C). A solution of tocilizumab, (0.1 g), was thoroughly dissolved in deionized water to create a stock solution with a concentration of 100 mg/L. The reactor was equipped with the PHILIPS TUV-PLL6W model, featuring Low-pressure, a maximum wavelength ranges of 385 nm and 6 W. A micro-diffuser was employed to introduce a consistent flow of ozone gas into the reaction medium by pumping gas that is high in oxygen through an ozone device (Oneteck, Iran). Regulating the O_3_ concentration by adjusting the flow rate of feed oxygen. The magnetic mixing was employed to homogenize the solution within the reactor. For the measurement of tocilizumab, 10 ml of the reaction mixture was taken from the reactor, passed through 0.22 m syringe filters, and finally injected into high-performance liquid chromatography (HPLC). The mean values were calculated by taking the average of the results from the three trials for each test, and the tocilizumab, TOC, and COD reduction rates in the O_3_-HPCP process were determined using (Eq. 4)^29^.

4 $$ {\text{Efficency removal}}(\% ) = \left[ {\frac{{{\text{C}}_{0} - C_{t} }}{{C_{0} }}} \right] \times 100 $$

The quantity of tocilizumab, COD, and TOC (mg/L) that is present initially and after a certain point in time is denoted by C_o_ and C_t_, respectively.

### The variables optimization of O_3_-HPCP based on CCD

The CCD approach within response surface methodology was employed in order to optimize the O3-HPCP for the tocilizumab degradation. Table 1 presents an overview of the various variables within a specified range.

**Table 1:** The range of Factors on Performance of process.

Factor	Name	Units	Minimum	Maximum	Coded low	Coded high	Mean	Std. Dev
A	pH	–	2.0	10.0	− 1 ↔ 4.0	+ 1 ↔ 8.0	6.0	+ 1/81
B	MOFs dose	mg/l	0.1250	0.625	− 1 ↔ 0.25	+ 1 ↔ 0.5	0.375	+ 0/11
C	O_3_ Concentration	mg/l-min	10.0	50.0	− 1 ↔ 20.0	+ 1 ↔ 40.0	30.0	+ 9/04
D	Temziva Concentration	mg/l	5.0	25.0	− 1 ↔ 10.0	+ 1 ↔ 20.0	15.0	+ 4/52
E	Degradation Time	min	15.0	75.0	− 1 ↔ 30.0	+ 1 ↔ 60.0	45.0	+ 13/55

The design of the CCD served as the foundation for the fifty trials documented in Table S.1, with the intention of tocilizumab degradation.

The representation of the tocilizumab degradation through O_3_-HPCP can be expressed through the utilization of a mathematical model known as a second-order polynomial model (Eq. 5)^31^:

5 $$ Y. = \beta_{0} + \sum\limits_{i = 1}^{k} {\beta_{i} } x_{i} + \sum\limits_{i = 1}^{k} {\beta_{ii} } x_{i}^{2} + \sum\limits_{i = 1}^{k - 1} {\sum\limits_{j = i + 1}^{k} {\beta_{ij} } } x_{i} x_{j} + \varepsilon $$

Tocilizumab removal (%) is represented by Y., the intercept is β_0_, the linear, quadratic, and interaction effect coefficients of the variables are β_i_, β_ii_, and β_ij_, respectively, the coded testing classes of the variables are xi and x_j_, the number of independent variables is k, and the residual error is e. To allow comparisons between components with differing units, an encoding of each variable's value was calculated using (Eq. 6).

6 $$ x_{i} = \frac{{X_{i} - X_{0} }}{\Delta x} $$

where the coded value of the variable is X_i_, ∆_x_ which is the difference between the high and low values of the variable, X_0_ represents the low value of the variable. Analysis of variance (ANOVA) was used to find the interaction between response and variables using P-values and F-values. The correlation coefficients are R^2^ and R^2^_adj_. Also, R^2^ predicts were applied.

Under ideal circumstances, an analysis was carried out on the effects of different light sources (visible light, UV-A, and UV-C), the rate of the kinetic reaction, the impact of synergistic effect, variations in wavelength scanning, and energy consumption.

### Analytical methods

After each trial, a sample was gathered and filtered with a 0.45 µ micropure-filter and subsequently injected into the HPLC. A HPLC system with a fluorescence detector was utilized to measure the tocilizumab concentration. Utilization of the FF‐C18 non-porous column, which was packed with 2 μm particles (50 mm × 3.2 mm inner diameter, Kyoto, Japan) was performed. The mobile phase A was formulated with 0.1% trifluoroacetic acid (TFA), while solvent B was composed of 30% isopropanol, 70% acetonitrile, and 0.1%. Following a linear elution of A/B (90:10) to A/B (70:30) for one min, a linear elution of A/B (70:30) to A/B (55:45) for six min, a linear elution of A/B (55:45) to A/B (5:95) for two min, a linear elution of A/B (95:5) to A/B (0:100) for one min, and a final isocratic elution of A/B (90:10) for one min was utilized to create the gradient profile. 0.5 mL/min was established as the rate of flow for the mobile phase, while the column temperature was fixed at 75 °C^32^. The TOC analysis was conducted using Analytikjena's Multi C/N 3100 TOC Analyzer. As outlined in the conventional approach, COD levels were measured through titrimetric analysis (5220-C; closed-reflux)^33^.

### Supplementary investigation

#### Reaction kinetics and synergic effect

The decay of tocilizumab in the ideal circumstances of the O_3_-HPCP procedure was demonstrated to follow a first-order reaction (Eq. 7)^34^.

7 $$ {\text{ln}}(C_{t} /C_{0} ) = \, - k_{ppa} t $$

In the context, C_t_ represents the residual concentration, C_0_ represents the initial concentration of tocilizumab (mg/L). The variable t denotes the reaction time (min), and k_app_ denotes the rate constant (1/min).

An evaluation of the synergistic effect was conducted under ideal conditions of parameters determined by (Eq. 8)^35^.

8 $$\mathrm{Synergist effect }=\frac{Performance\, of\, O3-HPCP \,process(\%)}{Adsorption+photolysis+simple\, ozonation (\%)}$$

#### Reusability of catalyst, quenching test and anions effect

Six experiments were conducted to assess the reusability of catalyst and durability of the process under the best O_3_-HPCP conditions. The reaction of free radicals with organic radical scavengers and the effect of anions were investigated under ideal conditions.

#### Electrical energy demand

The electricity energy operating (EEO) of the O_3_-HPCP under ideal conditions as determined by (Eq. 9)^36^.

9 $$EEO=\frac{38.4*P}{V*Kobs}$$

The power consumption, P (kWh), and the volume of treated solution, V (L), can be found.

### Ethical statement

The above study has been approved by the Ethics Committee of the Baqiyatallah University of Medical Sciences (Code: IR.BMSU.BLC.1402.025).

## Results and discussion

### Characters of catalyst

#### XRD

**Figure 2:**
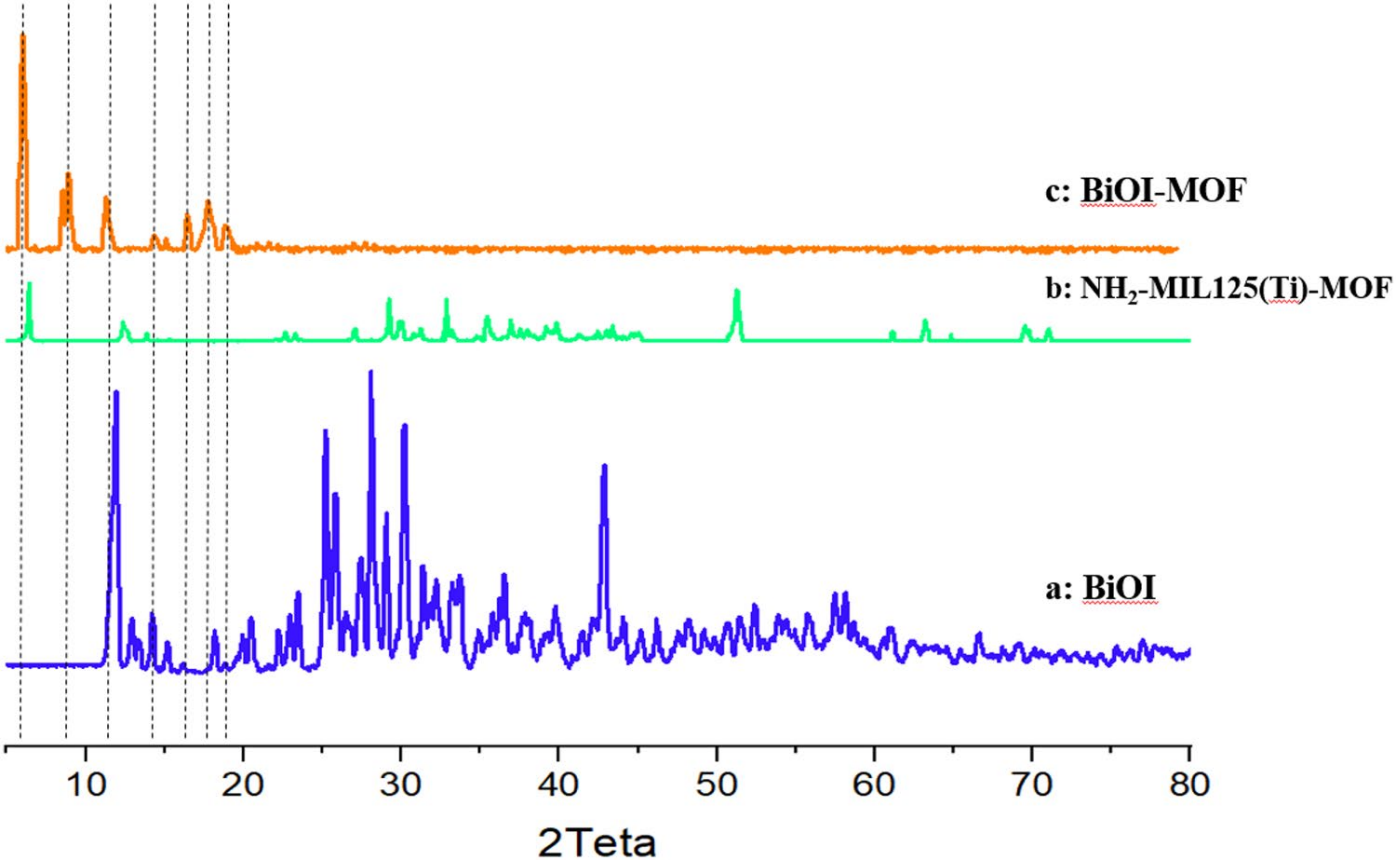
The XRD patterns of BiOI, MOF, and BiOI-MOF.

Figure 2 represent the X-ray diffraction patterns of BiOI, NH_2_-MIL125, and BiOI-MOF samples. BiOI with four major peaks illustrated in (Fig. 2a). These peaks numbered (102), (110), (200), and (212). All diffraction peaks from synthesized BiOI (JCPDS no. 10–0445) indicated a tetragonal phase. There indicates high crystallinity through the sharp and distinct peaks. Our previous study, shows the similar results^24^. In (Fig. 2b) presented XRD patterns of MOF. The standard pattern revealed MOF peaks at 6.8°, 9.7°, 11.8°, 15.7°, 16.5°, 17.8°, 19.1°, and 19.5°. Analysis of XRD patterns indicates that the orthorhombic phase has a correlation to lattice parameters of a = 14.89 Å, b = 5.9 Å, and c = 18.95 Å. By utilizing BDC and NH_2_BDC as a connecting agent, it becomes possible to generate three-dimensional pores within a sequence of corner-sharing TiO_6_. A comparable findings was reported by Mehralipour and their associates^37^. Finally, the BiOI-MOF nanocomposite structure exhibits certain peaks, similar to the ones observed in the pure BiOI and MOF, as demonstrated in (Fig. 2c). There is a significant change in the positions and strength of the peaks. The XRD results aligned with those of Du and co-workers' study^38^. Using Scherrer's equation (Eq. 1), the dimensions of the crystalline structures in the BiOI, MOF, and BiOI-MOF specimens were ascertained to measure 26/5, 47/8, and 37/1 nm, correspondingly.

#### FT-IR

**Figure 3:**
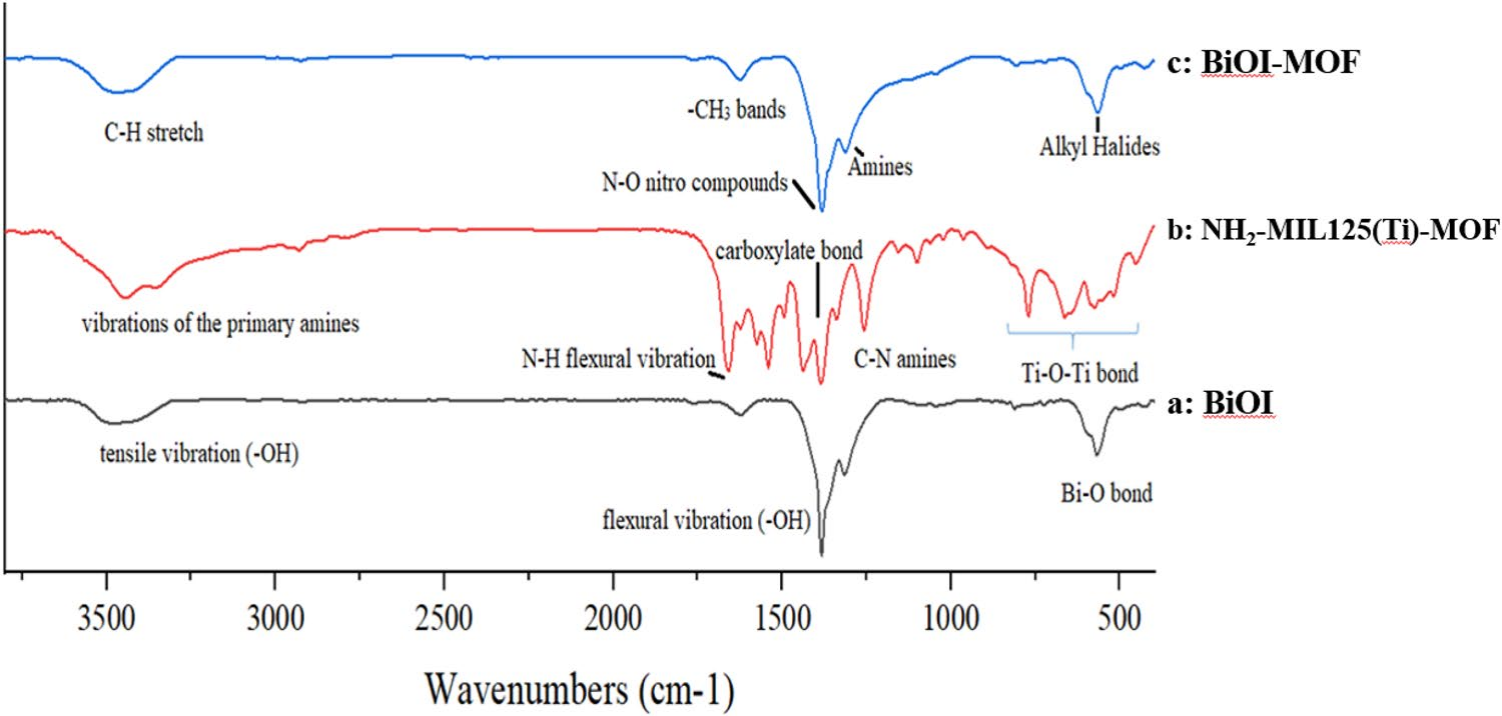
The Fourier Transform Infrared Spectroscopy of BiOI, MOF, and BiOI-MOF.

FTIR spectroscopy was utilized to examine the organic moieties of the samples (Fig. 3).

The main peaks appeared at 570.1, 1311.8, 1383.5, 1628.1, and 3532.4 cm^−1^ in pure BiOI. The vibrational frequency of the A_2_u type Bi-O bond is symmetrical at 570.1 cm^−1^. The BiOI demonstrates a powerful adsorption between 1300–1700 cm^−1^, along with a prominent peak in the range of 3185 cm^−1^ to 3478 cm^−1^ that is caused by the flexural (-OH) and tensile vibrations (-OH) of isolated water molecules on the BiOI's surface. Results that matched those reported by Vahabirad and his team^39^. The interval from 500 to 1500 cm^−1^ observed in MOF displayed distinctive attributes commonly associated with organic compounds, as illustrated in (Fig. 3b). The 1255 cm^−^1 and 1622 cm^−1^ peaks can be utilized to ascertain the typical tensile strength of aromatic N–H flexural vibrations and C-N amines, correspondingly. The obtained results indicate the presence of two distinct peaks at 1535 cm^−1^ and 1433 cm^−1^, thereby providing evidence for the existence of the carboxylate bond. Additionally, the absorption peaks that appear at 450 cm^−1^ are linked to the customary vibrations of Ti–O-Ti bonds. Both symmetric and asymmetric tensile vibrations of primary amines were observed to exhibit extensive peaks at approximately 4000 cm^−1^. Zhao and colleagues reported significant peaks at 773, 1258, 1385, 1539, 1662, 2524, 3059, 3348, and 3450 cm^−1^^40^. A depiction of the FT-IR spectrum of the BiOI-MOF can be observed in (Fig. 3 c). The highest peaks of the spectrum were identified at 565.5, 807, 1313, 1382, 1624, and 3464 cm^−1^. Amine groups, alkyl halides, -CH_3_ signals, N–O nitro compounds, C-H stretching, and C-H bands can be identified at the peaks. Studies conducted by Han and Du and their teams of researchers verified the heterojunction between BiOI at NH_2_ with NH_2_-MIL125(Ti)^27,38^.

#### Morphology analysis

**Figure 4:**
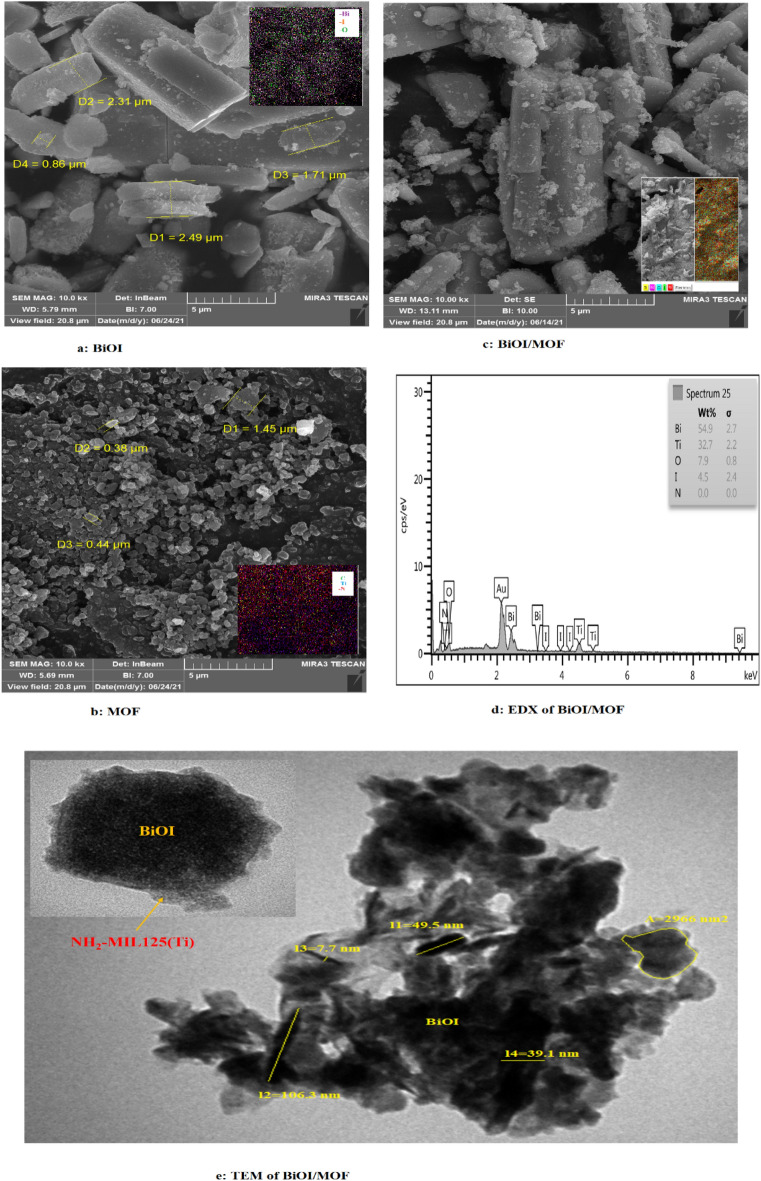
Scanning Electron Microscopy of (**a**) pure BiOI, (**b**) pure MOF, (**c**) BiOI-MOF composite, (**d**) Energy Dispersive X-ray Spectroscopy of BiOI-MOF composite, (**e**) BiOI-MOF composite TEM.

The FESEM, EDX, and EDS mapping technique applied to analyze the samples (Fig. 4). The morphology of thickness plate structures is demonstrated in (Fig. 4a), which is a BiOI FESEM image. In comparison to a synthesized structure, it has a longer length-to-width ratio. Arumugam and coworkers employed an alternate solvent in their research. A nano-sheet formation with a square-like structure was discerned when water (H_2_O-BiOI) was used as a solvent^41^. The morphology of BiOI is affected by the synthesis methods, chemicals, and solvents that are utilized. The constructed MOF has a thin, disk-like shape (Fig. 4b). The structure of a MOF can be altered by adjusting the organic-metal ratio. Findings have suggested that MOF structures transform from rectangular to round as organic linkers increase. The uniformity of the nanoplate morphology of NH2-MIL125(Ti) was observed in Zhang and coworkers' study^42^. BiOI-MOF is depicted in (Fig. 4c) as a rod with small particles. The structure of BiOI-MOF was comparable to Du and colleagues' analysis^43^. Furthermore, (Fig. 4d) displays the EDX image of BiOI. The samples show varying proportions of the primary components of each structure. The TEM image, depicted in (Fig. 4e), illustrates the effective dispersion of the NH_2_-MIL125(Ti) composite onto the BiOI surface, resulting in the formation of a core–shell architecture. Additional information regarding the structure of the BiOI, MOF, and BiOI-MOF catalysts is presented in (Figs. S1–S3) to enhance comprehension.

#### BET analysis

**Figure 5:**
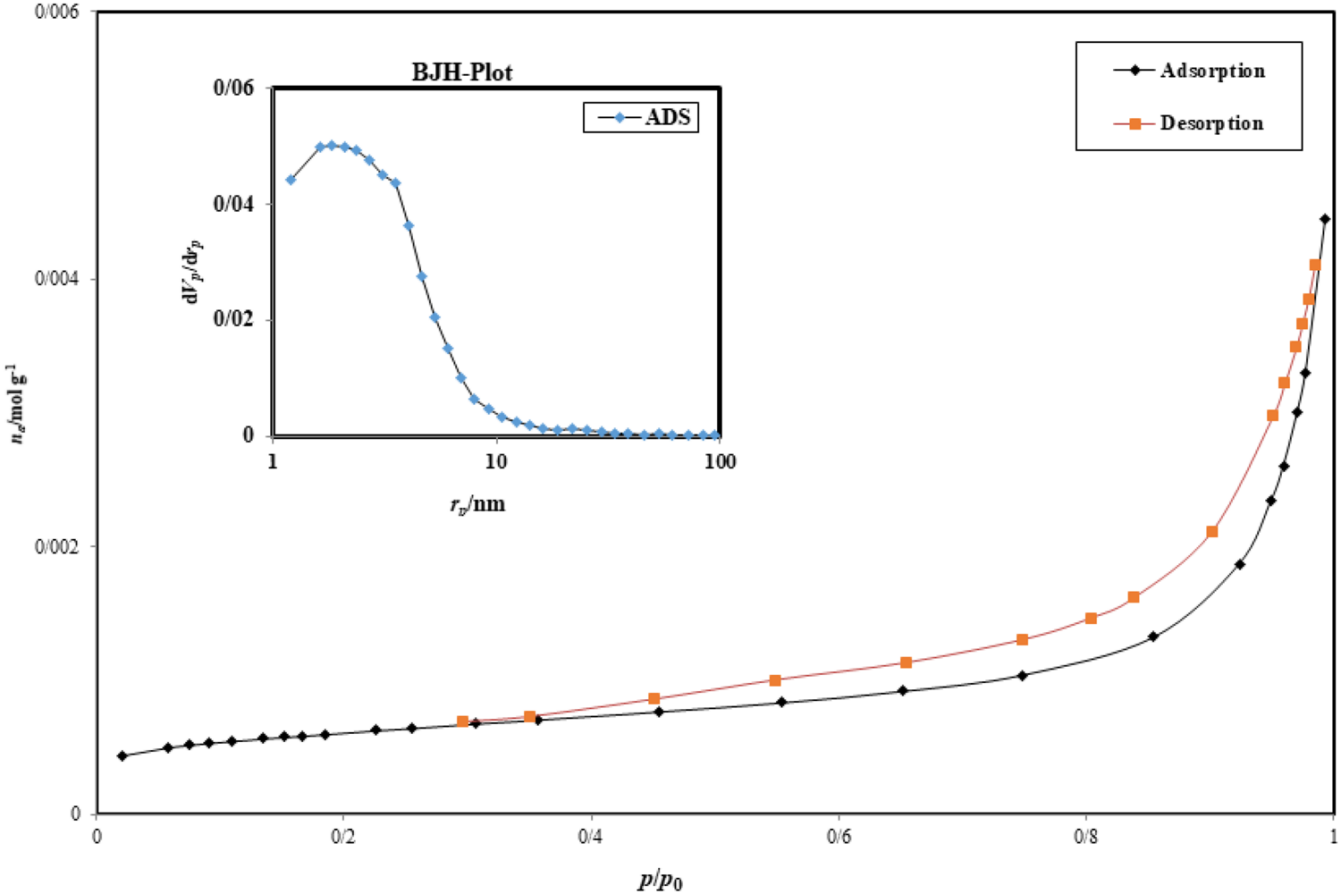
BiOI-MOF Pilot involving absorption and desorption.

Figure 5 displays the nitrogen adsorption–desorption isotherms for BiOI-MOF. The relationship between pressure and adsorption/desorption rate is significant. The hysteresis is classified as H3 and type IV due to its curved shape. This particular type of hysteresis is present in a non-hard cavity that has been cut into a certain shape. The tensile strength effect has a strong slope and is also known as the H_3_ hysteresis repulsion branch. The precursor's BET surface area and pore volume were determined using the N2 adsorption–desorption isotherm and BJH techniques, and were found to be 939.92 m^2^/g and 16.17 cm^3^/g, respectively.

#### UV–vis analysis

**Figure 6:**
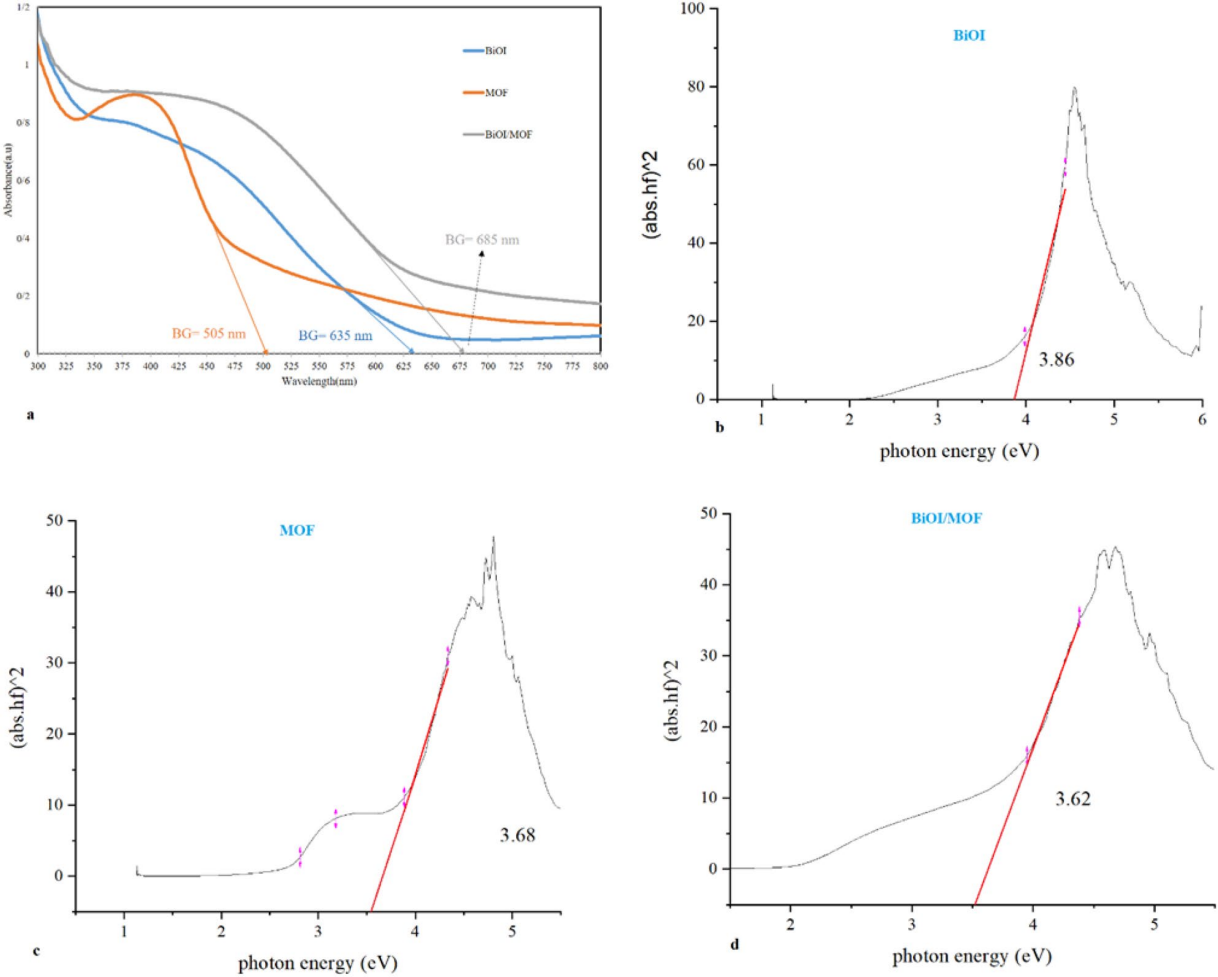
(**a**) Ultraviolet–Visible spectroscopy and, (**b**–**d**) Diffuse Reflectance Spectroscopy analysis of BiOI, MOF, and BiOI-MOF.

The UV–visible spectrum is displayed in (Fig. 6). The boundary line of BiOI, MOF and BiOI-MOF are located at 635, 505 and 685 nm respectively. The analysis reveals that BiOI-MOF has a greater band edge than either BiOI or MOF independently. Since the band gap has been found to be within the visible light spectrum, it can be concluded that the catalyst can be activated by visible light. Moreover, the DRS analysis revealed that the main catalyst has a narrower band gap against BiOI and MOF and uses low energy for activation. When compared to BiOI and MOF, the BiOI-MOF's structure has changed, as evidenced by the alterations made to the boundary line and diffuse reflectance. At 685 nm, the range line has grown. Furthermore, the band gap is now 3.62 eV. These modifications may have an impact on the catalyst's electrical characteristics and enhance its capacity for charge separation and photocatalysis. Comparable findings were reported in our earlier research^21^.

#### XPS analysis

**Figure 7:**
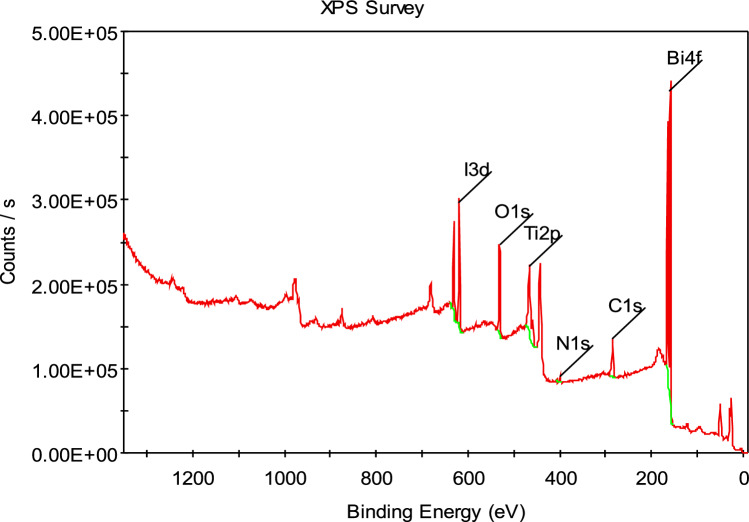
XPS spectra of BiOI-MOF.

XPS (Fig. 7) was used to evaluate the chemical state and surface composition of BiOI-MOF. The XPS spectrum affirms the components of Bi, I, Ti, O and C elements. Additionally, the purification of BiOI and NH_2_-MIL125(Ti) is verified. Table S1 summarizes the BiOI-MOF components. Jiang and their colleagues obtained similar outcomes^44^.

By analyzing the XRD results, which include changes in the spectrum such as peak removal, formation of new peaks, shift in peak positions, and changes in peak intensity, as well as analyzing the morphology of the BiOI-MOF catalyst in comparison to BiOI and NH_2_-MIL125(Ti), conducting EDS elemental mapping analysis to determine the presence of elements on the catalyst surface, analyzing the weight percentage and element ratio in EDS analysis, and confirming the presence of Ti, Bi, N, C, and I elements through XPS analysis, it can be concluded that the heterogeneous connection between BiOI and NH_2_-MIL125(Ti) has been successfully achieved and the BiOI-MOF catalyst has been synthesized correctly.

### O_3_-HPCP activity

#### Statistical analysis and the creation of a CCD

The results of tocilizumab degradation were statistically evaluated in order to develop a response surface model and determine the ideal circumstances for O_3_-HPCP. (Table S2). For identifying the data deviation factors in an ANOVA study, the F-value and P-value are trustworthy statistical markers. A significant statistical model with a high F-value and a low p-value (≤ 0.05) was chosen as a result of these indexes. A quadratic model would be the most appropriate to fit the relationship between the anticipated and experimental values of tocilizumab, according to Fisher's F-test (Table S3).

Utilizing ANOVA, the data for tocilizumab degradation (Table S4) exhibited a high level of reliability and a significantly low probability value for the quadratic regression model, indicating its strong capability to effectively describe the patterns within the real data and predicted values. This model proved to be highly accurate and skilled in forecasting responses, as evidenced by the correlation coefficients (R^2^, R^2^_adj_, and R^2^ predict). A strong correlation exists among R2, R2adj, and R2 predict, demonstrating the model's ability to make accurate predictions^45,46^. The F-value for the degradation of tocilizumab has been calculated to be 347.93 and the P-value (0.0001) strongly indicate a successful fit between the experimental and expected response values. An Adequate Precision ratio of greater than 4.0 indicates an acceptable model. The equation for the percentage of tocilizumab removal was expressed in terms of coded factors as follows:

10 $$  \begin{aligned} & {\text{tocilizumab removal }}\left( \% \right) \, = \, - {161}.{3 } + { 29}.{2} \times {\text{pH }} + { 295}.{8} \times {\text{MOFs }} + { 2}.{6} \times {\text{O}}_{{3}} \\ & \quad - {1}.{6} \times {\text{ Temziva Concentration }}  + { 1}.{9} \times {\text{Time }} - { 4}.{3} \times {\text{pH}} \times {\text{ MOFs }} \\ & \quad - \, 0.00{8} \times {\text{pH}} \times {\text{O}}_{{3}} - \, 0.0{3} \times {\text{pH}} \times {\text{Temziva Concentration}} + \, 0.0{1} \times {\text{pH}} \times {\text{Time }} \\ & \quad + \, 0.{6} \times {\text{MOFs}} \times {\text{O}}_{{3}} + { 1}.{3} \times {\text{MOFs}} \times {\text{Temziva Concentration }} + \, 0.{1} \times {\text{MOFs}} \times {\text{Time }} \\ & \quad + \, 0.0{3} \times {\text{O}}_{{3}} \times {\text{Temziva Concentration }}  - 0.000{1} \times {\text{O}}_{{3}} \times {\text{Time }} \\ & \quad - 0.00{7} \times {\text{Temziva Concentration}} \times {\text{Time }} - { 2}.{3} \times {\text{pH}}^{2} \, - { 355}.{88} \times {\text{MOFs}}^{2} \, \\ & \quad - 0.0{4} \times {{\text{O}}_{{3}}}^{2} \,  - 0.0{2} \times {\text{Temziva Concentration}}^{2} - 0.0{1} \times {\text{Time}}^{2} \\ \end{aligned} $$

The removal of tocilizumab was affected by the studied mediators, as outlined in (Eq. 10). The quadratic model showed a CV lower than 10%, confirming the accuracy and repeatability of the experiments. Furthermore, it was established that all interactions between the variables were of significance. Statistical analysis indicated that there was a statistically significant correlation between variables and the removal of tocilizumab.

#### The influence of factors on the tocilizumab degradation from O_3_-HPCP

##### pH of solution

**Figure 8:**
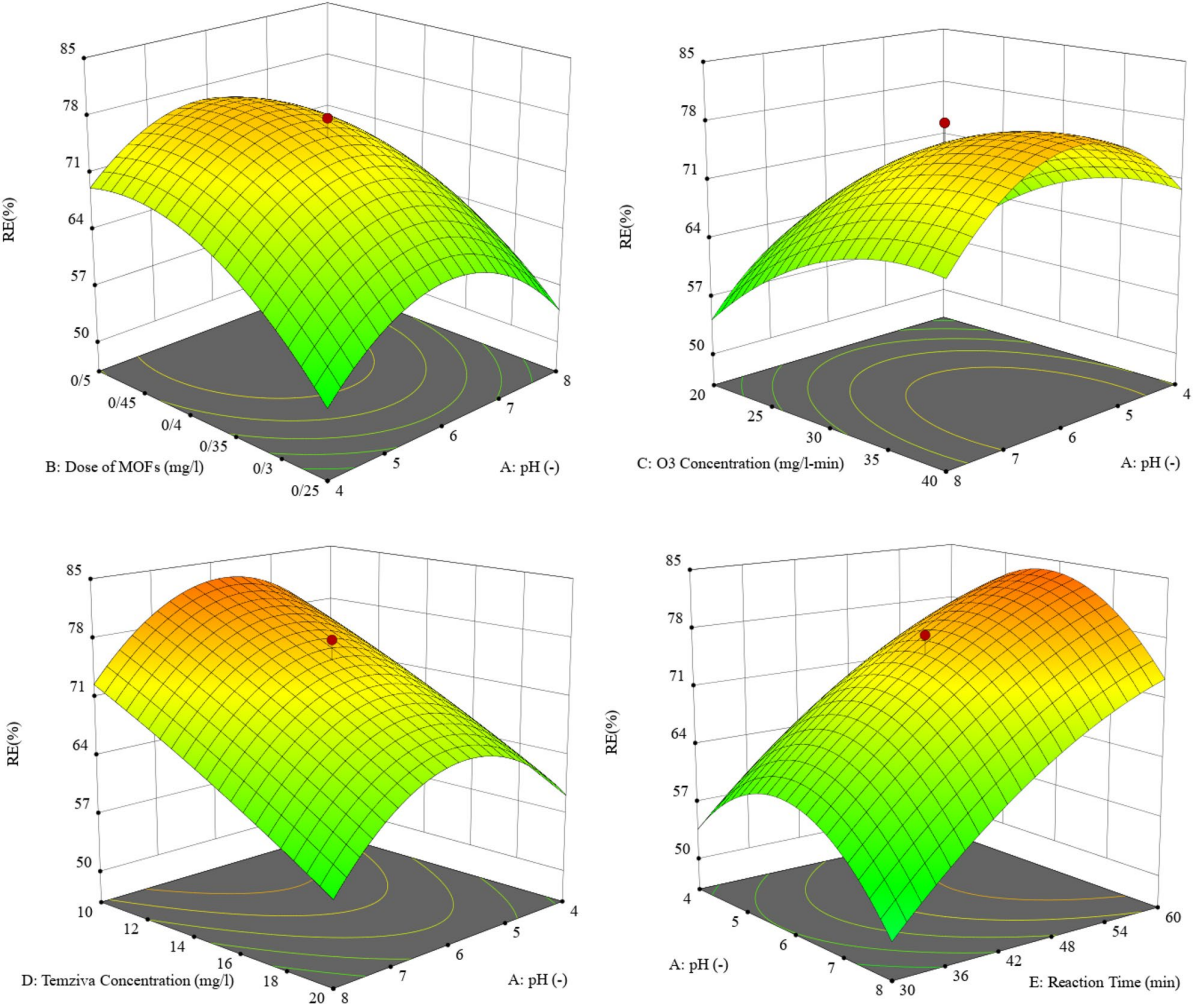
Investigation of the variables' influence on the removal of tocilizumab in the O_3_-HPCP method.

The efficiency of O_3_-HPCP is notably impacted by the solution's pH. The outcomes of varying pH levels (ranging from 4 to 8) on the efficiency of tocilizumab removal are depicted in Fig. 8. As the pH of the solution increased, there was a significant enhancement in the removal efficiency of tocilizumab, reaching its peak efficacy at slightly acidic pH levels, approximately around 6. The pH of the solution plays a role in several processes, including the hydrolysis of pollutants, the ionization of pollutants, the characteristics of the catalyst surface, the activity of oxidants and reactive species, as well as the degradation pathway. Also, zero-point charge pH (pH_zpc_) of catalyst and pKa of tocilizumab have an important role in efficiency degradation of pollutant. The most effective degradation of tocilizumab appears to be when the pH is at the (pH_zpc_) of 6.3 for BiOI-MOF. If the pH is below 6.3, the photocatalyst has a positive charge on its surface, but when the pH is above 6.3 it has a negative charge. Due to the pKa of tocilizumab being 6.8, a more acidic pH than that of the zero-point charge encourages the adsorption of tocilizumab molecules on the photocatalyst surface, leading to an enhanced degradation of the photocatalyst in weakly acidic conditions. An elevation in pH levels results in heightened coulombic repulsion between the negatively charged surface of BiOI-MOF and the OH^−^ species involved in the photocatalytic oxidation process. This, in turn, diminishes the efficiency of degradation. The pH can influence electrostatic interactions between functional groups of the catalyst, such as ionization in this scenario^47^. Ozone breaks down fast into free radicals at higher pH levels, which have a far greater speed and efficiency of breaking down organic materials than ozone molecules^48^. It was noted that the pH_zpc_ value of the photocatalyst measured 6.3, thereby indicating that the surfaces of the photocatalyst possess the ability to act as protonating and non-protonating agents in lower and higher pHzpc ranges, respectively.

Subsequently, upon the reduction of the pH level, minimal adsorption was observed as a result of the electrostatic repulsion between the catalyst, which carried a positive charge, and the tocilizumab molecules that had undergone protonation. Several studies have showed that the decomposition of contaminants typically occurs through direct and indirect oxidation mechanism during an ozonation process. The direct reaction of tocilizumab and ozone is observed at lower pH levels. As the pH of the solution increased, there was an increase in the rate of ozone decomposition and the production of free radicals, accompanied by the generation of free radicals with a high potential for oxidation. These radicals have a greater capability for tocilizumab decomposition. The molecules of tocilizumab can interact indirectly with free radicals in a basic environment, leading to improved removal performance. Asgari et al. investigated the capability of the photocatalytic ozonation process to removal of ceftazide. According to this study, a pH value of 11.0 was the most desirable pH of the solution^49^. Similarly, Jonathan C. Espíndola et al. reported findings of their own^50^.

##### Dose of catalyst

The quantity of the catalyst is a critical parameter that impacts heterogeneous-AOPs that are based on the catalyst. The research conducted in this study investigated various amounts of BiOI-MOF to determine the effectiveness of O_3_-HPCP in removing tocilizumab (Fig. 8). Results demonstrated that the removal efficacy of tocilizumab was notably improved when the catalyst dose increased from 0.25 to 0.5 mg/l. In spite of this, catalyst dosages of 0.46 mg/l did not show any noteworthy differences. A higher level of catalyst dose had an unfavorable effect on O_3_-HPCP. Generally, this effect is caused by particles amassing and clustering together, leading to a decline of surface active sites. When there is too much catalyst in the solution, UV light can be diffused, which prevents ozone and UV radiation from reaching the catalyst surface. The surface area of the active site for photocatalytic activity can be increased by adding more catalyst. Because of this, the nanoparticles had a higher level of ozone adhesion, leading to the generation of more active radicals due to ozone destruction. This resulted in an increase of tocilizumab removal effectiveness. In addition, oxygen radicals are produced when nanoparticles and ozone are combined in an alkaline environment. Oxygen radicals in water stimulate the formation of ^·^OH radicals, which increases ozonation efficiency^48^. Other studies of photocatalytic ozonation have come to similar conclusions, which are consistent with this study. Lu and their colleagues conducted an investigation of tetracycline hydrochloride decomposition through photocatalytic ozonation. This study used a catalyst of 0.0 to 0.8 g/l of Bi_2_WO_6_. The process had an efficiency of 78% when the dose was 0.5 g/l, however within 120 min this dropped to 65% when it reached 0.8 g/l^51^. Results consistent with Yu et al.'s study were discovered^52^.

##### Ozone concentration

The most significant economic factor in O_3_-HPCP is the amount of inlet ozone. This factor is strongly connected to energy utilization. The range of ozone gas concentration studied in this study was from 20 to 40 mM/l-min (Fig. 8). According to the findings, the process's efficiency increased somewhat when the ozone concentration was changed from 20 to 30 mMol/l-min. Increased ozone concentrations cause the rate of mass transfer in the reaction medium to increase. As the rate of ozone gas flow increases, the concentration of dissolved ozone in the solution rises. Reactive oxygen species, especially hydroxyl radicals, generate in greater quantities as a result of the synergistic impact. Adsorption of dissolved ozone molecules by photocatalysts is made easy due to the weak hydrogen bonds with their surface hydroxyl groups. Electron capture on the surface of the photocatalyst results in the formation of anions of ozonide radicals. Therefore, the ozonide radicals (O_3_^•−^) heighten the level of ^·^OH radicals and the potency of tocilizumab degradation^51^. In contrast, if the air flow coming in is too much, and the rate of moving from gas to liquid is slow, the ability to remove will be decreased. In general, the kind of procedure, kind of reaction reactor, type of pollutant, and specifications of the intermediate chemicals all affect the amount of ozone required for O_3_-HPCP^53^. Previous studies have reported various concentrations of ozone as being optimal. The optimal ozone concentration was reported by Heydari et al.^54^ at 11 mg/l, while Yu et al.^55^ reported 1.5 mg/l-min.

##### Reaction time

Another important component influencing heterogeneous AOPs is reaction time. This research explored the effect of reaction time on the removal of tocilizumab in the timeframe of 30 to 60 min (Fig. 8). There was a marked improvement in degradation effectiveness for tocilizumab with increasing reaction time. As previously noted, O_3_-HPCP remove tocilizumab by two distinct oxidation pathways. Ozone molecules, photolysis and free radicals as well as electron–hole pairs created can all contribute to pollutant destruction through indirect and direct oxidation. The reaction time was increased, leading to complete pollutant destruction and mineralization with a decreased number of intermediate compounds. Consequently, the ideal reaction time depends on the kind of pollutant, qualities of the reaction reactor, other operational variables and conditions. Various reaction time have been highlighted in prior investigations. Lu et al.^51^, report 120 min as the optimal time.

The optimal value of experiment parameters for tocilizumab degradation by O_3_-HPCP was ultimately determined by us using the Design-Expert software. The ideal conditions for process were pH of the solution 7.0, catalyst dose of 0.46 mg/l, reaction time of 59 min, concentration of 32 mMol/l-min, and initial tocilizumab concentration of 10 mg/l. Here, tocilizumab was degraded 92%.

#### Synergist effect, recyclability of photocatalyst, COD and TOC test

**Figure 9:**
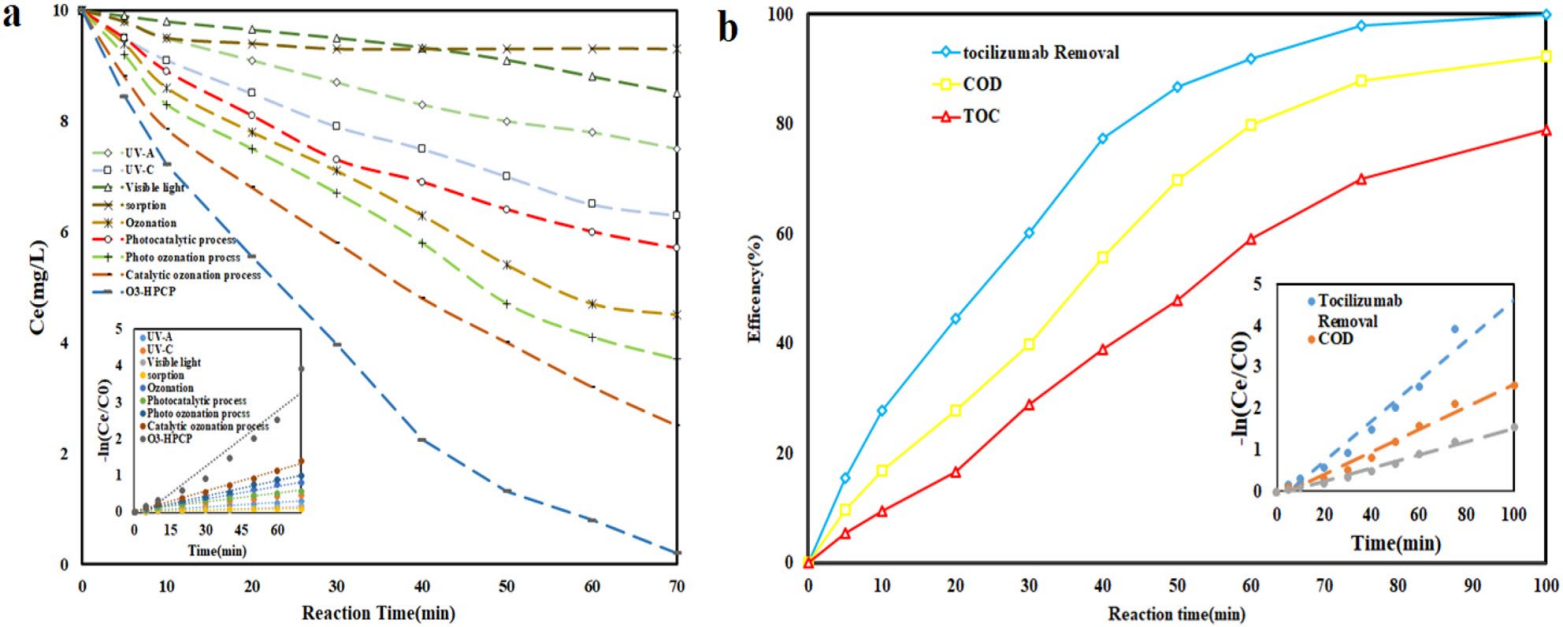
(**a**) Synergistic effect of mechanisms in O_3_-HPCP. (**b**) COD and TOC reduction in O_3_-HPCP in optimum condition (pH = 7.0, Catalyst dose = 0.46 mg/l, time = 60 min, O_3_ = 32 mMol/l-min, Tocilizumab = 10 mg/l).

Results from (Fig. 9a) demonstrate the functionality of single mechanisms, dual mechanisms and O_3_-HPCP in the degradation of tocilizumab under optimal circumstances. According to the findings, sorption (6.9%), ozonation (53%) and photolysis (UV-C = 35%, UV-A = 22, and visible light = 12%) are the three main mechanisms by which tocilizumab degrades. These mechanisms lack sufficient potential. The dual processes of catalytic ozonation (68%), photocatalyst (40%), and photo ozonation (59%) were found to be more successful in degrading tocilizumab than the individual processes. Ultimately, the O_3_-HPCP could remove 92% of the tocilizumab. The findings support the synergistic effect between a variety of mechanisms, leading to a more active degradation of tocilizumab based on ROS_s_. By following (Eq. 8), the SF coefficient yielded a value of 1.22. Photolysis and ozonation were utilized to degrade tocilizumab through direct oxidation. Oxidation with a direct process has limited capacity because of its selectivity. Consequently, the pollutant removal efficiency is evidently inadequate. However, in binary processes and O_3_-HPCP, oxidation is done indirectly through ROS_s_. These radicals have a capacity of high oxidation for organic pollutants, such as tocilizumab, due to their non-selective properties. When light is directed towards a catalyst in photocatalytic reactions, holes develop in the valence band (VB) and electrons form in the conduction band (CB). Since the catalyst's CB potential is higher than the reaction medium's redox potential for oxygen and oxygen peroxide, electrons are transported to the oxygen and abundantly transformed to oxygen. ROS generation and the use of photogenerated carriers will affect the photocatalytic system^56^. Electrophilic ozone, in comparison to direct ozonation, can be easily used in photocatalytic systems to trap photogenerated electrons. The photogenerated electron of the catalyst is efficiently transferred, causing the electron–hole pairs to be effectively separated when exposed to simulated light irradiation, resulting in a high production of ROS. The ozone utilization ratio can be improved through the use of a catalyst in the photo ozonation process. An increase in ozone during the reaction will create more ROS, which will be helpful regarding the mineralization of organic compounds. The combination of photocatalysis and ozonation produces more ROS than when each process is used separately, making it effective in the removal of organic pollutants^57^. In Lu et al.^51^ study, TOC removal in O_3_, O_3_/light and O_3_/light/catalyst were 18, 20 and 78% respectively. As illustrated in (Fig. 9b), the findings of tocilizumab removal, and reduction of COD, and TOC are presented. It has been found out by the results that the COD and TOC reduction rate is lower than that of tocilizumab removal. Tocilizumab, COD, and TOC reduced by 92, 79.8, and 59%, respectively, under ideal circumstances. The current discrepancy demonstrates the generation of organic byproducts and intermediates. The results showed that increasing the reaction time improves COD and TOC reduction. Over 90% of COD and roughly 80% of TOC were decreased in just 100 min. Asgari et al.^48^ reported 100, 92.3 and 81.1% CIP, COD and TOC removal respectively in 60 min.

**Figure 10:**
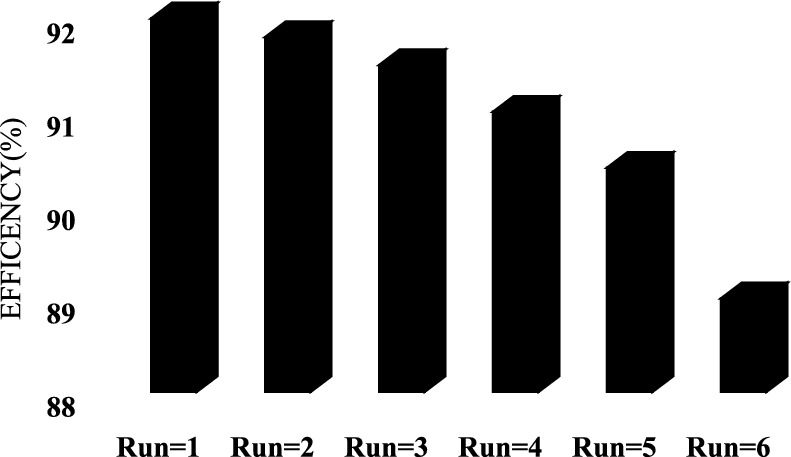
Test of catalyst recycling in the O_3_-HPCP (pH = 7.0, Catalyst dose = 0.46 mg/l, time = 60 min, O_3_ = 32 mMol/l-min, Tocilizumab = 10 mg/l).

The essential characteristics of catalysts for practical applications encompass the capacity to be recycled and utilized again. The (Fig. 10) illustrated the capacity of the photocatalyst to be recycled was investigated over the course of six consecutive cycles in ideal circumstances. The catalyst was taken out of the solution and allowed to dehydrate at 80 °C for 24 h after each cycle. After six consecutive cycles, the removal of tocilizumab demonstrated a 3% reduction. The O_3_-HPCP efficiency somewhat declined, indicating that catalysts could be recycled for a minimum of six cycles. The increasing obstruction of photocatalytic adsorption sites by tocilizumab and co-products, the use of active oxidizing species by intermediates, and the ongoing washing and drying processes might all be contributing factors to the small decline in catalyst activity^49^. Bagheri et al.^58^ observed that after eight iterative cycles of catalyst, there was a 92% efficiency in dye degradation.

#### Quenching tests and ions influence on O_3_-HPCP performance

**Figure 11:**
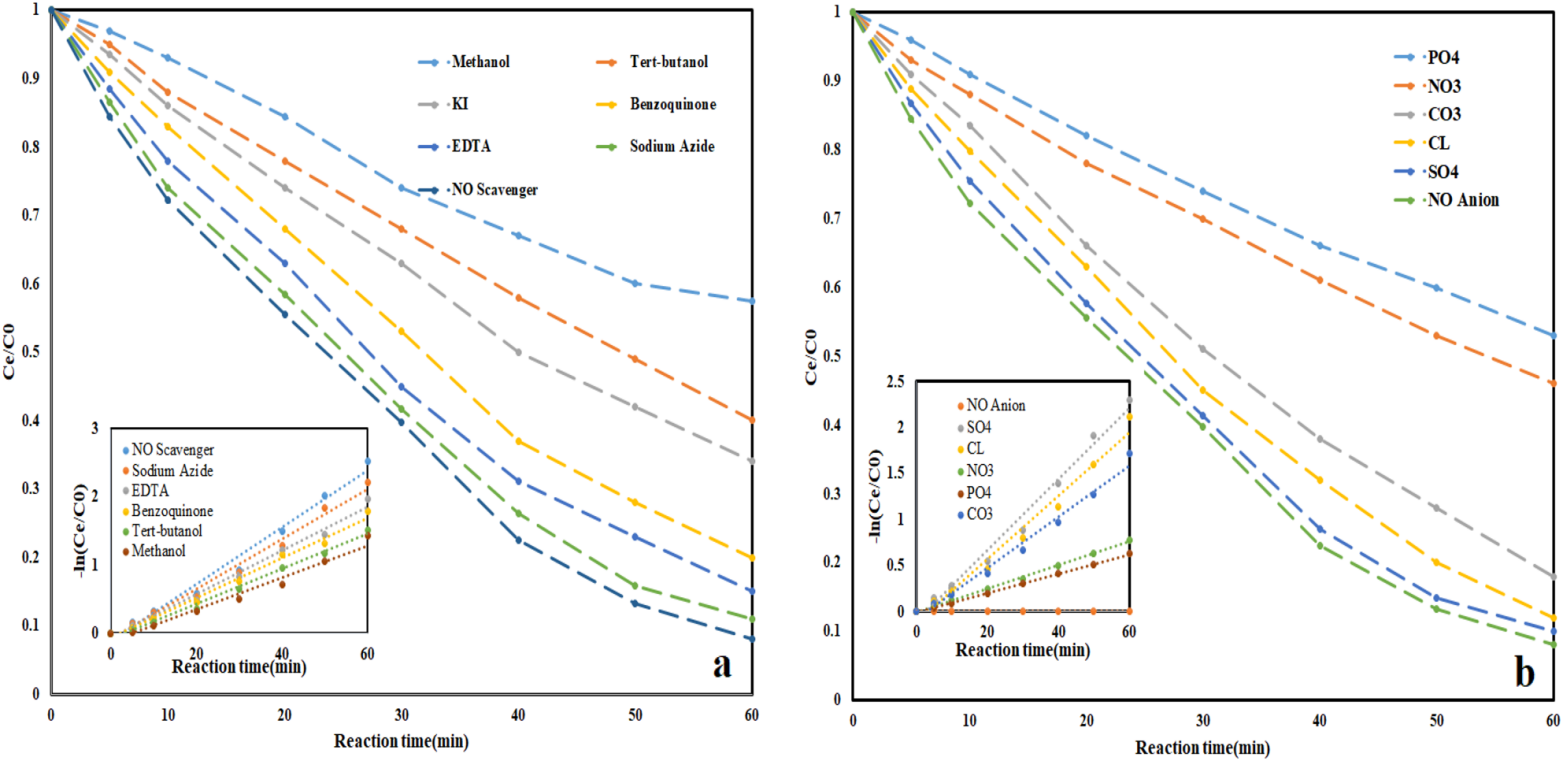
(**a**) Effect of radical scavengers (**b**) ions on tocilizumab removal in O_3_-HPCP (pH = 7.0, Catalyst dose = 0.46 mg/l, time = 60 min, O_3_ = 32 mMol/l-min, Tocilizumab = 10 mg/l).

The results of the quenching test are presented in (Fig. 11a). Tert-butanol, KI, methanol, EDTA, sodium azide, and benzoquinone were chosen as some ROS_s_ scavengers. The results indicated that methanol had the most effect on O_3_-HPCP performance in tocilizumab removal and the lowest effect on performance observed in the sodium azide presence. Because reactive species are responsible for the degradation of organic pollutants in AOPs, it is essential to understand the principles of AOP_s_ and the process of persistent organic pollution degradation by examining the creation, identification, and reaction mechanism of ROS_s_ in AOP_s_. There are different methods like electron spin resonance (ESR), transient absorption spectrum, quenching test and HPLC for identification. One indirect way to determine the reactive species in AOP_s_ is to perform the quenching test. The foundation of quenching tests is on the variations in the reactivity and reaction rates between a specific scavenger and a potentially reactive species. The selectivity of the scavenger is a crucial factor in the quenching experiments^59^. ROS_s_ in O_3_-HPCP include OH^·^, O_2_^·−^, HO_2_^·−^, O_3_^·−^ and e^−^–h^+^ pairs. So, methanol, tert-butanol, KI, benzoquinone, EDTA, and sodium azide were chosen as a α-Hydrogen OH^·^, OH^·^, surface OH^·^, O_2_^·−^, O_2_^1^ and O_3_^·−^, and e^−^ -h^+^ pairs scavengers, respectively. The results revealed that the OH^·^ plays the most important role in pollutant oxidation and the e^−^–h^+^ pair has the least effect. The α-hydrogen OH^·^ mechanism involves the attack of the highly reactive OH^·^ on the α-carbon, which is situated adjacent to a functional group, in organic compounds. This interaction leads to the degradation of the organic pollutants. Hydroxyl radicals are commonly generated through processes such as the photolysis of hydrogen peroxide under UV light or through direct reaction with ozone. These hydroxyl radicals exhibit strong reactivity and possess potent oxidizing capabilities. Subsequently, the abstraction of the α-hydrogen by the hydroxyl radical generates a carbon-centered radical within the organic pollutant. This radical is also highly reactive and capable of initiating a cascade of reactions. Furthermore, the carbon-centered radical can react with molecular oxygen to yield peroxy radicals (ROO^·^). These peroxy radicals can subsequently engage in further reactions with other organic molecules, thereby instigating a chain reaction that degrades the organic pollutant into smaller, less harmful fragments. Ultimately, the chain reaction persists until termination reactions take place. Termination reactions typically involve the recombination of radicals or their reaction with other reactive species, effectively concluding the degradation process^60,61^. In Bashiri et al.^62^, and Li and et al.^63^ studies similar results have been reported.

Practical wastewater contains several inorganic ions. Subsequently, it is necessary to investigate how distinct inorganic ions can impact O_3_-HPCP capability to tocilizumab removal. PO_4_^3−^, NO_3_^−^, CO_3_^2−^, Cl^−^, and SO_4_^2−^ with 0.1 mM concentration were chosen and the influence on the O_3_-HPCP performance is presented in (Fig. 11b). The reaction of inorganic anions and reactive species is the primary factor of inorganic anions, enabling the formation of a novel ROSs. Since these ROS_s_ have smaller ORP than OH^·^, the procedure is less efficient. With the existence of SO_4_^2^, SO_4_^·−^ generated that has 2.5 eV ORP, and the efficiency is less affected (Eq. 11).

11 $$ {\text{SO}}_{{4}}^{{{2} - }} + {\text{OH}}^{ \cdot } \to {\text{SO}}_{{4}}^{ \cdot - } + {\text{ OH}}^{ - } $$

In the presence of Cl^−^ ion, the following reactions (Eqs. 12 and 13) occur and cause the formation of Cl^·^ (ORP = 2.03 eV), and because it is weaker than OH^·^, it causes a decrease in the process's efficiency.

12 $$ {\text{Cl}}^{ - } + {\text{OH}}^{ \cdot } \to {\text{ClOH}}^{ \cdot - } $$

13 $$ {\text{ClOH}}^{ \cdot - } + {\text{H}}^{ + } \to {\text{Cl}}^{ \cdot } + {\text{ H}}_{{2}} {\text{O}} $$

In the presence of CO_3_^2−^ ion, the following reaction (Eq. 14) occur and cause the formation of CO_3_^·−^ (ORP = 1.7 eV), and because it is weaker than OH^·^, it causes a decrease in the process's efficiency.

14 $$ {\text{CO}}_{{3}}^{{{2} - }} + {\text{OH}}^{ \cdot } \to {\text{CO}}_{{3}}^{ \cdot - } + {\text{ OH}}^{ - } $$

In the presence of NO_3_^−^ ion, the following reaction (Eq. 15) occur and cause the formation of NO_3_^·^ (ORP = 2.3–2.5 eV), and because it is selective and weaker than OH^·^, it causes a decrease in the process's efficiency.

15 $$ {\text{NO}}_{{3}}^{ - } + {\text{OH}}^{ \cdot } \to {\text{NO}}_{{3}}^{ \cdot } + {\text{ OH}}^{ - } $$

In the presence of PO_3_^4−^ ion, the following reaction (Eq. 16) occur and cause the formation of PO_4_^2·−^ (ORP = 1.5 eV), and because it is weaker than OH^·^, it causes a decrease in the process's efficiency.

16 $$ {\text{PO}}_{{3}}^{{{4} - }} + {\text{OH}}^{ \cdot } \to {\text{PO}}_{{4}}^{{{2} \cdot - }} + {\text{ OH}}^{ - } $$

Analysis of prior studies showed that researchers have examined different anions. Yuan and et al.^64^ and Saleh and et al.^65^ reported similar reports in owner studies.

#### Reaction kinetic and electrical energy demand

**Figure 12:**
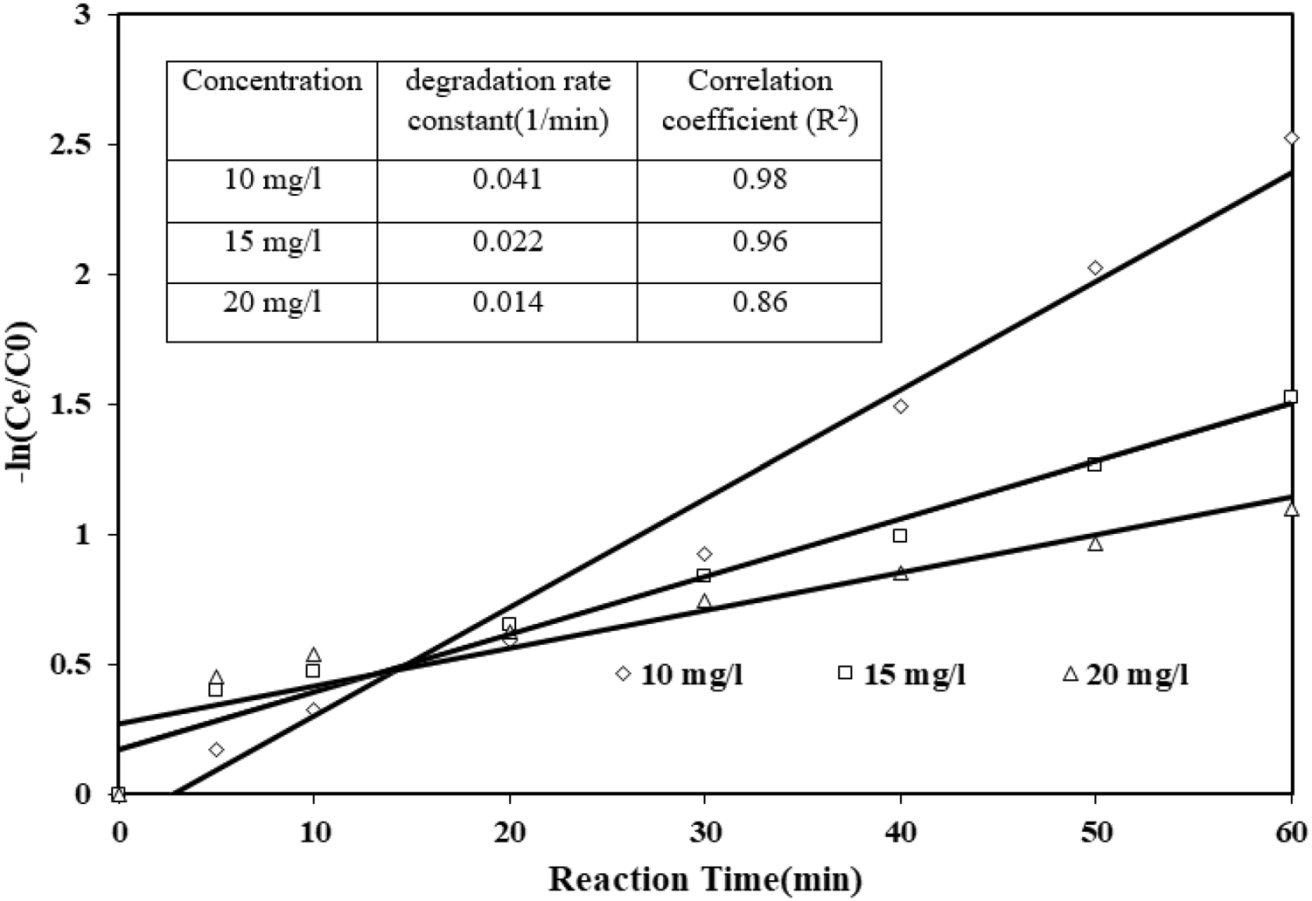
Tocilizumab degradation kinetics in O3-HPCP (pH = 7.0, Catalyst dose = 0.46 mg/l, time = 60 min, O3 = 32 mMol/l-min, Tocilizumab = 10 mg/l).

The first-order model (Eq. 7) illustrated the rate of tocilizumab degeneration over O_3_-HPCP in optimal conditions. The degradation of tocilizumab in 10, 15, and 20 mg/l concentrations was showed in (Fig. 12) over 60 min using a linear first-order kinetic model. The first-order model indicates that the degradation of tocilizumab in O_3_-HPCP is accurately reflected by the experimental data (R^2^ > 0.98) when the initial concentration is 10 mg/l. Through the evaluation of prior research, it has been found out that first-order kinetics is frequently observed in most AOPs^66,67^.

Energy consumption must be factored into practical applications. The ozone generator and the UV lamp were the source of EEO in this process. EEO in different processes is presented based on Eq. 9 in Table 2.

**Table 2:** EEC in different processes in optimum condition (pH = 7.0, DOC = 0.46 mg/l, RT = 60 min, O_3_ = 32 mMol/l-min, Tocilizumab = 10 mg/l).

Electric energy per order of pollutant removal	P_Electric_		Reaction time	Reactor volume	Log(C_0_/C_e_)	EE/O
	Ozone generator	UV lump				
Unit	kW		min	L		kW/h-m^3^-order^1^
*Processes*						
Photolysis	–	0.03	60	0.6	0.07	249.1
Ozonation	0.15				0.2	720.9
photocatalyst process		0.015			0.15	1126.4
UV/Ozonation	0.15	0.015			0.4	694.7
Catalytic ozonation	0.15				0.5	415.2
O3-HPCP	0.15	0.015			3	161.8

**Figure 13:**
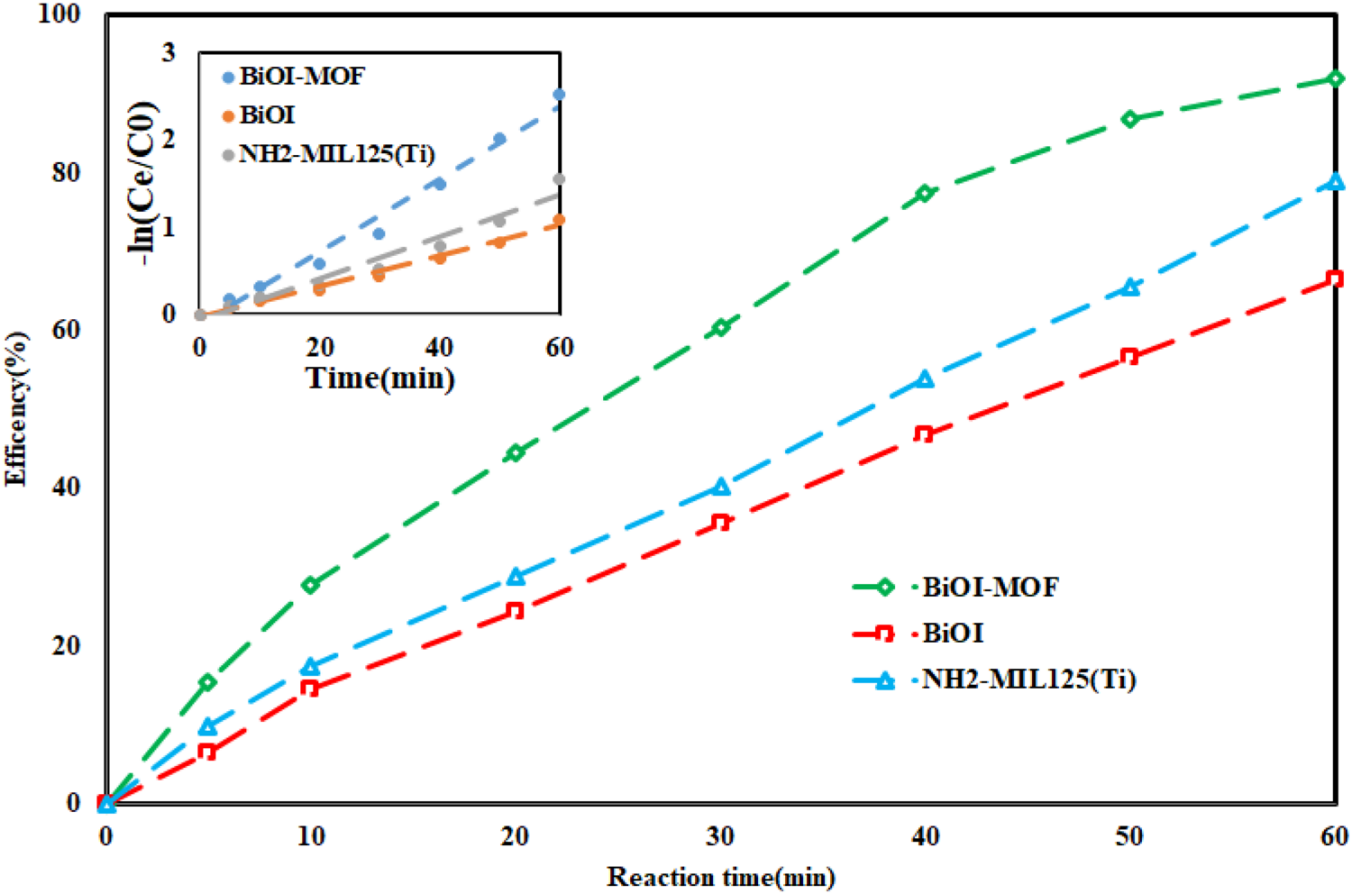
Degradation efficiency of tocilizumab under different catalysts (pH = 7.0, Catalyst dose = 0.46 mg/l, time = 60 min, O_3_ = 32 mMol/l-min, Tocilizumab = 10 mg/l).

The results show that in the O_3_-HPCP, based on tocilizumab removal, energy consumption is more appropriate.

Prior studies have demonstrated that the effectiveness of the EEO procedure is contingent upon several factors, including the ozone generator's power, the lamp's power, the reaction time, the reaction chamber's size, and the overall process efficiency^68^. The EEC recorded in Kang et al. research was 55 KWh/m^3^^23^.

#### catalyst components effect and mechanism of reactions

To compare the photocatalytic effectiveness of the BiOI-MOF component to that of the MOF (NH_2_-MIL125(Ti)) and BiOI components, the study was conducted under optimal conditions. In this section, the results showed (Fig. 13) that when the heteroconjection structure occurs; it improves the photocatalytic properties of the catalyst and increases the efficiency of the process. The surface properties, such as pore volume, active surface area, pollutant specific absorption rate, have been altered in the BiOI/MOF structure in comparison to the pure structures, as well as optical properties such as band gap, consequently leading to an improved efficiency. The performance of the procedure varied depending on whether MnWO_3_ or pure WO_3_ was present in the research by Yazdanbakhsh and colleagues^69^.

The O_3_-HPCP mechanism described by (Eq. 17–28)^23^.

17 $$ {\text{Photo semiconductors }} + {\text{ light }}\left( {{\text{UV}} - {\text{C}},{\text{ A and visible}}} \right) \, \to \, \left( {{\text{h}}^{ + } + {\text{ e}}^{ - } } \right){\text{ pairs}} $$

18 $$ {\text{O}}_{{{\text{3aq}}}} + {\text{ e}} - \, \to {\text{ O}}_{{{\text{3aq}}}}^{ \cdot - } $$

19 $$ {\text{O}}_{{{\text{3aq}}}}^{ \cdot - } + {\text{ H}}^{ + } \to {\text{ HO}}_{{3}}^{ \cdot - } $$

20 $$ {\text{HO}}_{{3}}^{ \cdot - } \to {\text{ O}}_{{2}} + {\text{ OH}}^{ \cdot } $$

21 $$ {\text{O}}_{{{\text{3aq}}}} + {\text{ H}}_{{2}} {\text{O }} + {\text{ light }} \to {\text{ H}}_{{2}} {\text{O}}_{{2}} + {\text{ O}}_{{2}} $$

22 $$ {\text{H}}_{{2}} {\text{O}}_{{2}} + {\text{ light }} \to {\text{ 2 OH}}^{ \cdot } $$

23 $$ {\text{H}}_{{2}} {\text{O}}_{{2}} + {\text{ O}}_{{{\text{3aq}}}} \to {\text{ 2 OH}}^{ \cdot } + {\text{ O}}_{{{\text{3aq}}}}^{ \cdot - } $$

24 $$ {\text{H}}_{{2}} {\text{O}}_{{2}} \to {\text{ OH}}_{{2}}^{ - } + {\text{ H}}^{ + } $$

25 $$ {\text{O}}_{{{\text{3aq}}}} + {\text{ OH}}_{{2}}^{ - } \to {\text{ O}}_{{{\text{3aq}}}}^{ \cdot - \, } {\text{ + OH}}_{{2}}^{ \cdot } $$

26 $$ {\text{OH}}_{{2}}^{ \cdot } \leftrightarrow {\text{ O}}_{{2}}^{ \cdot - } + {\text{ H}}^{ + } $$

27 $$ {\text{O}}_{{{\text{3aq}}}} + {\text{ O}}_{{2}}^{ \cdot - } \to {\text{ O}}_{{{\text{3aq}}}}^{ \cdot - } + {\text{ O}}_{{2}} $$

28 $$ {\text{Free radicals }} + {\text{ Pollutant }} \to {\text{ CO}}_{{2}} + {\text{ H}}_{{2}} {\text{O }} + {\text{ non - toxic inorganic compounds}} $$

At the first (Eq. 17), Electrons and holes are produced when exposed to UV radiation. Ozone molecules employ photogenerated electrons to produce O_3_^•−^ based on the process described in (Eq. 18). The media's oxygen absorbs electrons to create HO_2_^•^/O_2_^•^, which may then combine with ozone to produce O_3_^•−^. Equation 19 states that O_3_^•−^ combines with H^+^ to create HO_3_^•−^ and OH^•^ (Eq. 20). (Eq. 21) generates hydrogen peroxide (H_2_O_2_), which then reacts to create OH^•^ and O_3_^•−^. Furthermore, a series of chemical events occur, leading to the creation of various oxidizing radicals, based on the data shown in (Eqs. 22–27). This ultimately results in the contaminant degrading, as (Eq. 28) illustrates. The combination of BiOI and NH_2_-MIL125(Ti) in the BiOI-MOF catalyst results in a diverse interaction, which enhances the generation of charge under light and reduces the recombination of electron–hole pairs, as compared to the pure BiOI. The mechanism can be attributed to several factors like Heterostructure formation, type-II band alignment, band-edge positions, enhanced surface area and active sites and synergistic effects. These factors collectively promote efficient charge transfer, separation, and utilization, leading to enhanced photoelectrochemical performance. A heterostructure is formed by the combination of BiOI and NH_2_-MIL125(Ti), with the two materials being in close proximity to one other. This heterostructure facilitates efficient charge transfer and separation at the interface. When light is absorbed, electrons in both BiOI and NH_2_-MIL125(Ti) become excited. The heterostructure helps in the effective transport of these excited electrons from BiOI to NH_2_-MIL125(Ti). The energy band alignment between BiOI and NH_2_-MIL125(Ti) exhibits a mostly type-II characteristic. In this band alignment configuration, one material has a valence band maximum (VBM) that is positioned at a higher energy level than the conduction band minimum (CBM) of the other material. The staggered band alignment results in the presence of an inherent electric field at the interface, which facilitates the separation of charges and inhibits the recombination of electrons and holes. The energy levels of the valence and conduction bands in the BiOI-MOF structure undergo modifications in comparison to the pure BiOI. NH_2_-MIL125(Ti) can induce a shift in the energy levels of BiOI, causing the CBM to be positioned higher and the VBM to be positioned lower. This shift in energy levels can generate a beneficial energy gradient that facilitates the efficient separation and movement of charges. NH_2_-MIL125(Ti) is a MOF material characterized by its substantial surface area and numerous active sites. The incorporation of NH_2_-MIL125(Ti) into BiOI enhances the light absorption capacity and introduces supplementary locations for charge separation and transfer. The active sites within the MOF framework serve as trapping or catalytic centers, enabling the separation and use of photogenerated charges. The distinct characteristics and capabilities of each material can synergistically enhance the separation of charges and minimize recombination. NH_2_-MIL125(Ti) may have effective charge transport characteristics, whereas BiOI may have pronounced visible light absorption. The combined impacts of the two materials can greatly enhance the overall photoelectrochemical performance^70–72^.

## Conclusion

The analysis of BiOI-MOF via XRD, FTIR, FESEM, EDS, EDS elemental mapping, UV–vis, BET and XPS yielded significant findings of catalyst synthesis optimization. According to XRD analysis, the BiOI-MOF displays specific peaks that resemble those seen in the pure BiOI and MOF. However, there is a notable alteration in the locations and intensities of these peaks. The most prominent peaks in the FTIR spectra of the catalyst were observed at wavenumbers of 565.5, 807, 1313, 1382, 1624, and 3464 cm^−1^. The peaks can be used to identify amine groups, alkyl halides, –CH_3_ signals, N–O nitro compounds, C–H stretching, and C–H bands. The BiOI-MOF was observed in the FESEM as a rod-shaped structure composed of tiny particles. The samples exhibit diverse ratios of the fundamental constituents of each form. The TEM image demonstrates the successful distribution of the NH_2_-MIL125(Ti) composite onto the BiOI surface, leading to the creation of a core–shell structure. The hysteresis is categorized as H_3_ and type IV, according to the BET study. The measured values for the surface area and pore volume were 939.92 m^2^/g and 16.17 cm^3^/g, respectively. The relative boundary wavelengths for BiOI, MOF, and BiOI-MOF are 635, 505, and 685nm, respectively. Moreover, the band gap has decreased to 3.62 eV. These alterations can potentially affect the electrical properties of the catalyst and improve its ability to separate charges and facilitate photocatalysis. The XPS spectrum confirms the presence of the elements Bi, I, Ti, O, and C. Furthermore, the validation of the purification process for BiOI and NH_2_-MIL125(Ti) is confirmed. The F-value obtained from the ANOVA analysis for the degradation of tocilizumab is 347.93. The corresponding P-value (0.0001) indicates a highly significant match between the experimental and anticipated response values. The optimal parameters for the process were a solution pH of 7.0, a catalyst dose of 0.46 mg/l, a reaction time of 59 min, a concentration of 32 mMol/l-min, and an initial tocilizumab concentration of 10 mg/l. Here, the degradation of tocilizumab reached 92%. The primary degradation mechanisms of tocilizumab include sorption (6.9%), ozonation (53%), and photolysis (UV-C = 35%, UV-A = 22%, and visible light = 12%). These mechanisms are deficient in their potential. The combined methods of catalytic ozonation (68%), photocatalyst (40%), and photo ozonation (59%) shown more efficacy in degrading tocilizumab compared to the separate processes. COD and TOC decreasing under optimum condition was 79.8, and 59% respectively, that lower than tocilizumab removal. The catalyst could be used for six consecutive operations with acceptable results and a 3% reduction in efficiency. ROS_s_ in O_3_-HPCP include OH^·^, O_2_^·−^, HO_2_^·−^, O_3_^·−^ and e^−^–h^+^ pairs. So, methanol, tert-butanol, KI, benzoquinone, EDTA, and sodium azide were chosen as a α-Hydrogen OH^·^, OH^·^, surface OH^·^, O_2_^·−^, O_2_^1^ and O_3_^·−^, and e^−^ -h^+^ pairs scavengers, respectively. The results indicated that methanol had the most effect on O_3_-HPCP performance in tocilizumab removal and the lowest effect on performance observed in the sodium azide presence. Furthermore, the results revealed that the OH^·^ plays the most important role in pollutant oxidation and the e^−^–h^+^ pair has the least effect. SO_4_^2−^ had the lowest effect on the efficiency of the process, and NO_3_^−^ had most effect in anions, the process's efficiency was inhibited by organic scavengers. The rate of reaction follows a first-order equation (R^2^ = 0.98). EEO under optimum condition 161.8 KWh/m^3^-order calculated. The combination of BiOI and NH_2_-MIL125(Ti) in the BiOI-MOF catalyst results in a diverse interaction, which enhances the generation of charge under light and reduces the recombination of electron–hole pairs, as compared to the pure BiOI. The mechanism can be attributed to several factors like Heterostructure formation, type-II band alignment, band-edge positions, enhanced surface area and active sites and synergistic effects. Lastly, a comparison between the current study and earlier research is provided in Table 3.

**Table 3:** comparison between the current study and earlier research based on O_3_-HPCP.

Processes	pollutants	Optimum condition	Efficiency (%)	Authors and references
photocatalytic ozonation process—TiO_2_ magnetic nanoparticles	Ceftazide	CFT concentration = 10 mg/L, pH = 11, catalyst dosage = 1.0 g/L and ozone flow = 0.22 g/h and RT = 15 min	efficiency and mineralization of 100 was 75.5% were obtained	Asgari^49^
photocatalytic ozonation—Bi_2_WO_6_	Tetracycline hydrochloride	Bi_2_WO_6_ dose = 0.5 g/L, O_3_ concentration = 10 mg/L, pH = 3.8, time = 120 min	Over 80% TOC was removed	Lu^51^
photocatalytic ozonation/	Oxytetracycline	pH 8.0, CD = 0.34 mg/l, RT = 56 min and O_3_ concentration = 28.7 mN	96.2, 77.2, 64.2% of OTC, COD, and TOC were removed	Mehralipour^20^
photocatalytic ozonation—ZnO/ICLT nanocomposite	Furosemide (FRS)	O_3_ = 11 mg/L, FRS = 33.18 mg/L, dose = 0.27 g/L and time = 30 min	99.8% of FRS was removed	Heidari^54^
Heterogeneous photocatalytic ozonation-Bi_2_WO_6_/TiO_2_	sulfamethoxazole	catalyst dosage = 0.2 g/L, the ozone concentration = 1.5 mg/L, the SMX concentration = 10 mg/L, the pH = 5.25, and reaction time = 180 min	the removal rate of SMX was 97.1%	Zhang^47^
*O*_*3*_*-HPCP/Co-MOF*	tocilizumab	pH = 7.0, CD = 0.46 mg/l, RT = 59 min, and O_3_ concentration = 32 mMol/l	92, 79.8, and 59% of tocilizumab, COD and TOC were removed	*Current study*

### Supplementary Information

Supplementary Information.

## Data Availability

The datasets generated and analyzed during the current study available from the corresponding author on reasonable request.
