# Supplementary material for: Tocilizumab degradation via photo-catalytic ozonation process from aqueous
Source: Sci Rep. 2023 Dec 16;13:22402. doi: 10.1038/s41598-023-49290-z (PMC10725442; doi:10.1038/s41598-023-49290-z)
Supplement: Supplementary file 1 — Supplementary Information. [file 41598_2023_49290_MOESM1_ESM.docx]

***Table S 1: Characteristics and quantities of elements in BiOI-MOF***

| ***Name*** | ***Peak BE*** | ***FWHM eV*** | ***Area (P) CPS.eV*** | ***Weight %*** |
| --- | --- | --- | --- | --- |
| I3d | 619.29 | 2.55 | 690831.13 | 12.51 |
| Bi4f | 159.35 | 1.70 | 1772170.25 | 42.31 |
| O1s | 531.02 | 3.44 | 393125.12 | 12.28 |
| C1s | 285.07 | 3.11 | 169423.64 | 9.61 |
| Ti2p | 466.05 | 5.25 | 563521.87 | 22.31 |
| N1s | 399.61 | 1.19 | 22816.83 | 0.97 |

***Table S2: Runs of O_3_-HPCP into tocilizumab removal***

|  | Factor 1 | Factor 2 | Factor 3 | Factor 4 | Factor 5 | Response 1 |
| --- | --- | --- | --- | --- | --- | --- |
| Run | A:pH | B:Dose of MOFs | C:O3 Concentration | D:Temziva Concentration | E:Reaction Time | R1 |
|  | - | mg/l | mg/l-min | mg/l | min |  |
| 1 | 4 | 0/25 | 40 | 20 | 60 | 55 |
| 2 | 8 | 0/25 | 40 | 10 | 30 | 45 |
| 3 | 2 | 0/375 | 30 | 15 | 45 | 42/5 |
| 4 | 6 | 0/375 | 30 | 15 | 45 | 75 |
| 5 | 8 | 0/5 | 40 | 10 | 60 | 81/6 |
| 6 | 4 | 0/5 | 20 | 10 | 30 | 49/9 |
| 7 | 4 | 0/25 | 20 | 20 | 60 | 40 |
| 8 | 4 | 0/5 | 20 | 20 | 60 | 56/7 |
| 9 | 6 | 0/375 | 30 | 5 | 45 | 90/8 |
| 10 | 8 | 0/25 | 40 | 20 | 60 | 54/6 |
| 11 | 4 | 0/25 | 20 | 20 | 30 | 20 |
| 12 | 6 | 0/375 | 30 | 15 | 75 | 85/9 |
| 13 | 6 | 0/375 | 30 | 15 | 15 | 42/4 |
| 14 | 8 | 0/25 | 40 | 20 | 30 | 33/6 |
| 15 | 4 | 0/5 | 40 | 20 | 30 | 52/5 |
| 16 | 8 | 0/5 | 40 | 20 | 30 | 45/3 |
| 17 | 6 | 0/375 | 30 | 15 | 45 | 75 |
| 18 | 6 | 0/375 | 30 | 25 | 45 | 56/5 |
| 19 | 6 | 0/375 | 30 | 15 | 45 | 75 |
| 20 | 4 | 0/5 | 20 | 20 | 30 | 35 |
| 21 | 6 | 0/375 | 30 | 15 | 45 | 75 |
| 22 | 8 | 0/25 | 20 | 20 | 60 | 40/4 |
| 23 | 6 | 0/375 | 10 | 15 | 45 | 45/5 |
| 24 | 4 | 0/5 | 40 | 10 | 30 | 60 |
| 25 | 8 | 0/5 | 20 | 20 | 30 | 30/1 |
| 26 | 8 | 0/25 | 20 | 20 | 30 | 19/4 |
| 27 | 6 | 0/375 | 30 | 15 | 45 | 75 |
| 28 | 8 | 0/25 | 20 | 10 | 60 | 65 |
| 29 | 8 | 0/5 | 20 | 20 | 60 | 50 |
| 30 | 8 | 0/5 | 20 | 10 | 60 | 70/3 |
| 31 | 6 | 0/375 | 30 | 15 | 45 | 77/9 |
| 32 | 6 | 0/125 | 30 | 15 | 45 | 42/3 |
| 33 | 4 | 0/5 | 40 | 20 | 60 | 75 |
| 34 | 4 | 0/5 | 20 | 10 | 60 | 75 |
| 35 | 8 | 0/5 | 40 | 20 | 60 | 70 |
| 36 | 4 | 0/25 | 40 | 10 | 30 | 50 |
| 37 | 6 | 0/375 | 30 | 15 | 45 | 75 |
| 38 | 4 | 0/5 | 40 | 10 | 60 | 85 |
| 39 | 8 | 0/5 | 40 | 10 | 30 | 60 |
| 40 | 4 | 0/25 | 20 | 10 | 60 | 59/8 |
| 41 | 8 | 0/25 | 20 | 10 | 30 | 38/3 |
| 42 | 6 | 0/375 | 50 | 15 | 45 | 72/5 |
| 43 | 8 | 0/5 | 20 | 10 | 30 | 45 |
| 44 | 4 | 0/25 | 40 | 20 | 30 | 35 |
| 45 | 10 | 0/375 | 30 | 15 | 45 | 36/1 |
| 46 | 6 | 0/625 | 30 | 15 | 45 | 66/1 |
| 47 | 6 | 0/375 | 30 | 15 | 45 | 75 |
| 48 | 4 | 0/25 | 40 | 10 | 60 | 70 |
| 49 | 4 | 0/25 | 20 | 10 | 30 | 38/8 |
| 50 | 8 | 0/25 | 40 | 10 | 60 | 70 |

***Table S3: An overview of the model's functionality***

| **Source** | **Sequential p-value** | **Lack of Fit p-value** | **Adjusted R²** | **Predicted R²** |  |
| --- | --- | --- | --- | --- | --- |
| Linear | < 0.0001 | < 0.0001 | 0/6551 | 0/6370 |  |
| 2FI | 0/9991 | < 0.0001 | 0/5701 | 0/5525 |  |
| **Quadratic** | **< 0.0001** | **0/1047** | **0/9930** | **0/9859** | **Suggested** |
| Cubic | 0/5741 | 0/0585 | 0/9926 | 0/8950 | Aliased |

***Table S 4: ANOVA for a quadratic model in tocilizumab in O_3_-HPCP***

| **Source** | **Sum of Squares** | **df** | **Mean Square** | **F-value** | **p-value** |  |
| --- | --- | --- | --- | --- | --- | --- |
| **Model** | 15828/15 | 20 | 791/41 | 347/93 | < 0.0001 | significant |
| A-pH | 67/34 | 1 | 67/34 | 29/60 | < 0.0001 |  |
| B-Dose of MOFs | 1614/17 | 1 | 1614/17 | 709/64 | < 0.0001 |  |
| C-O3 Concentration | 1727/91 | 1 | 1727/91 | 759/64 | < 0.0001 |  |
| D-Temziva Concentration | 2555/20 | 1 | 2555/20 | 1123/34 | < 0.0001 |  |
| E-Reaction Time | 5006/41 | 1 | 5006/41 | 2200/96 | < 0.0001 |  |
| AB | 37/20 | 1 | 37/20 | 16/35 | 0/0004 |  |
| AC | 1/02 | 1 | 1/02 | 0/4464 | 0/5094 |  |
| AD | 4/88 | 1 | 4/88 | 2/15 | 0/1536 |  |
| AE | 3/06 | 1 | 3/06 | 1/35 | 0/2554 |  |
| BC | 20/96 | 1 | 20/96 | 9/22 | 0/0050 |  |
| BD | 22/28 | 1 | 22/28 | 9/79 | 0/0040 |  |
| BE | 3/85 | 1 | 3/85 | 1/69 | 0/2035 |  |
| CD | 77/81 | 1 | 77/81 | 34/21 | < 0.0001 |  |
| CE | 0/0253 | 1 | 0/0253 | 0/0111 | 0/9167 |  |
| DE | 11/16 | 1 | 11/16 | 4/91 | 0/0348 |  |
| A² | 2759/13 | 1 | 2759/13 | 1212/99 | < 0.0001 |  |
| B² | 989/46 | 1 | 989/46 | 434/99 | < 0.0001 |  |
| C² | 608/48 | 1 | 608/48 | 267/51 | < 0.0001 |  |
| D² | 15/60 | 1 | 15/60 | 6/86 | 0/0139 |  |
| E² | 302/21 | 1 | 302/21 | 132/86 | < 0.0001 |  |
| **Residual** | 65/96 | 29 | 2/27 |  |  |  |
| Lack of Fit | 58/61 | 22 | 2/66 | 2/53 | 0/1047 | not significant |
| Pure Error | 7/36 | 7 | 1/05 |  |  |  |
| **Cor Total** | 15894/12 | 49 |  |  |  |  |


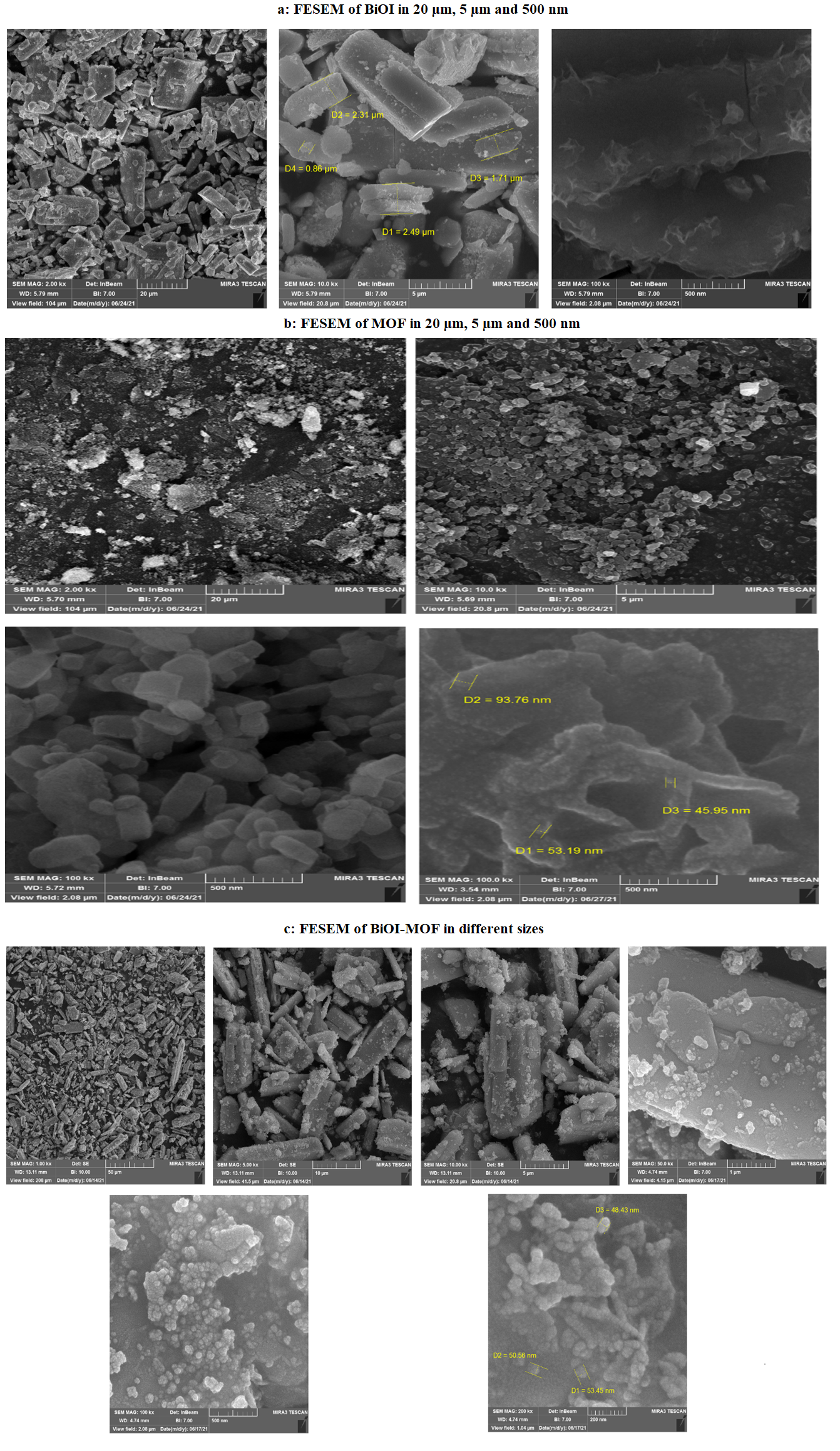


***Fig. S1: FESEM morphology analysis. a) BiOI, b) MOF, c) BiOI@MOF***

***
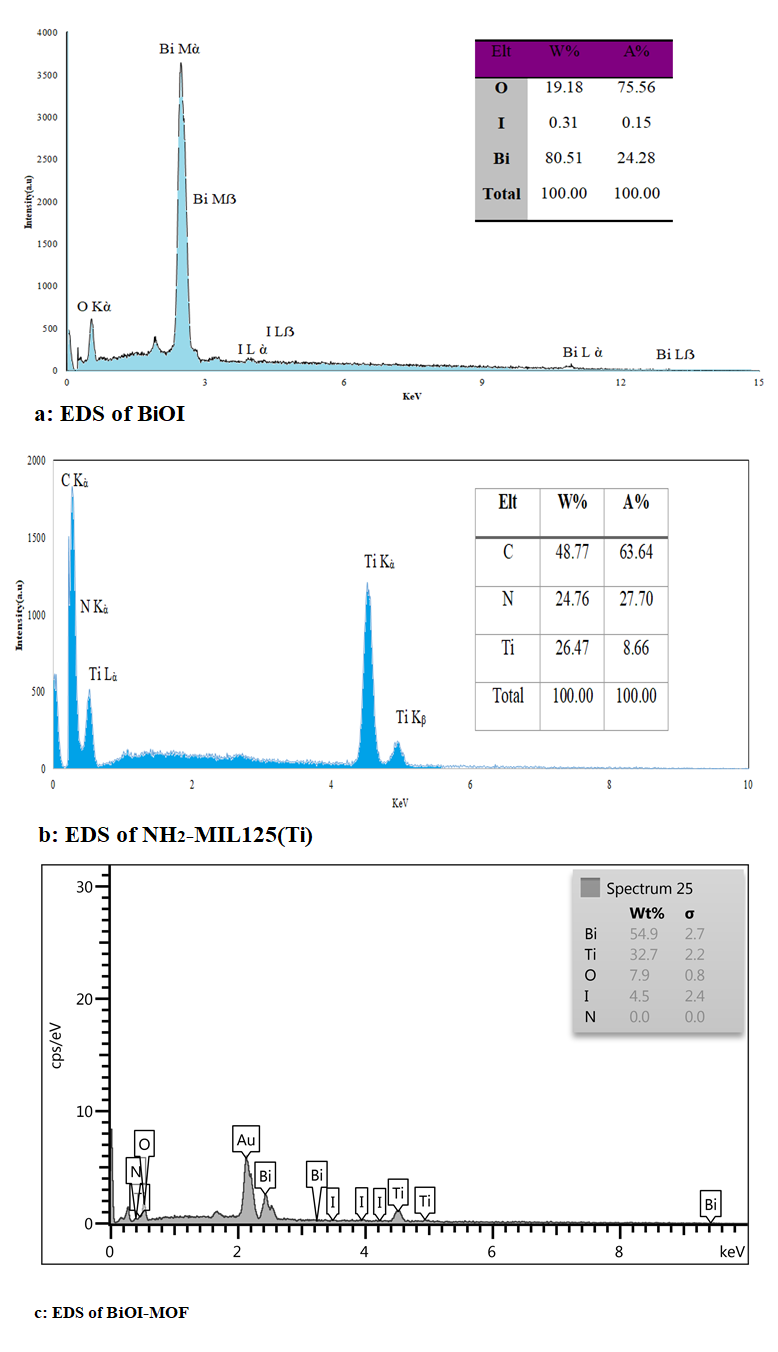
***

***Fig. S2: EDS analysis of a) BiOI, b) NH_2_-MIL125(Ti), c) BiOI-MOF***

***
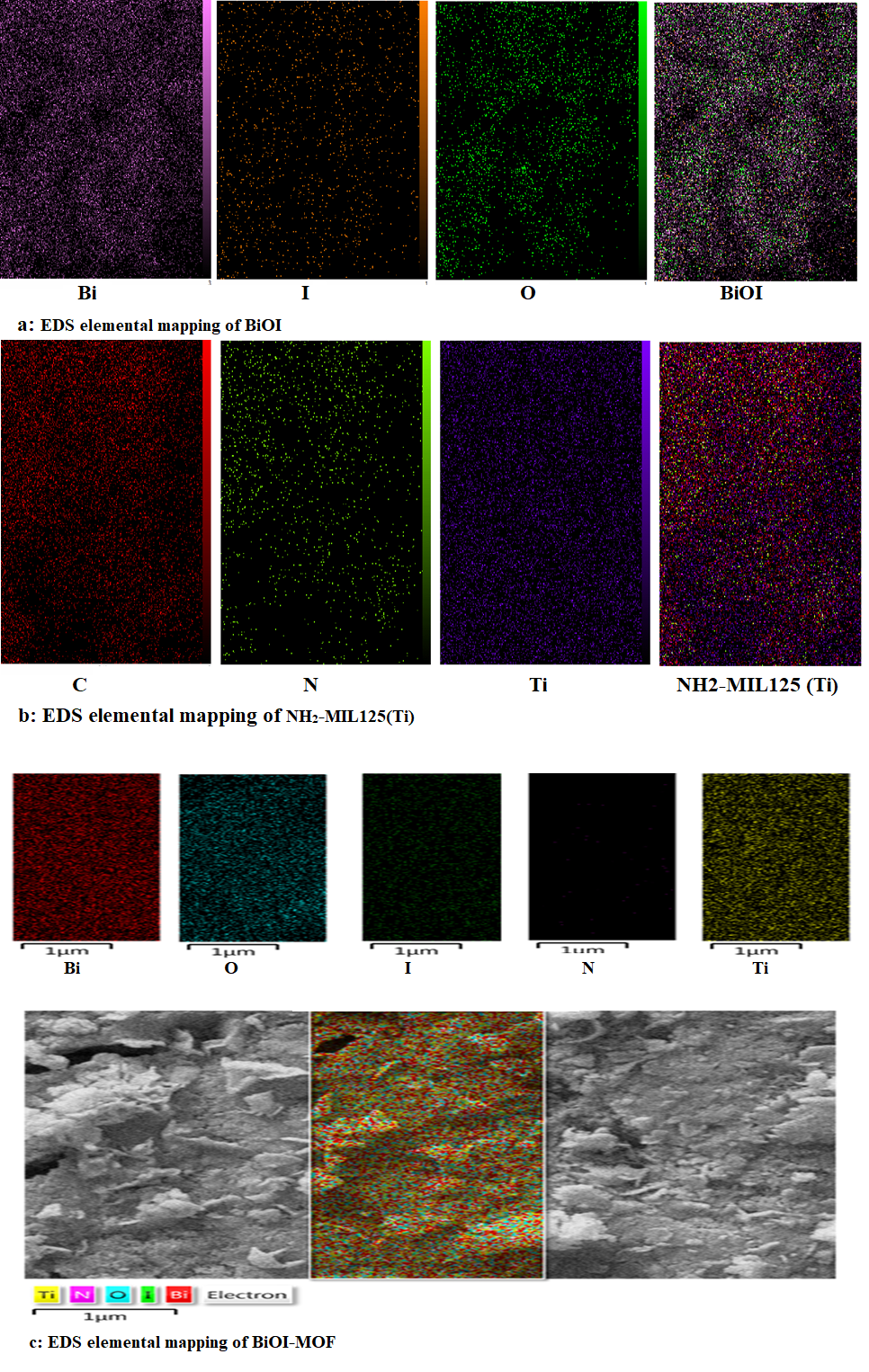
***

***Fig. S3: EDS elemental mapping analysis of a) BiOI, b) NH_2_-MIL125(Ti), c) BiOI-MOF***
